# Polymers and Related Composites via Anionic Ring-Opening Polymerization of Lactams: Recent Developments and Future Trends

**DOI:** 10.3390/polym10040357

**Published:** 2018-03-22

**Authors:** Tatyana Ageyeva, Ilya Sibikin, József Karger-Kocsis

**Affiliations:** Department of Polymer Engineering, Faculty of Mechanical Engineering, Budapest University of Technology and Economics, Műegyetem rkp. 3, 1111 Budapest, Hungary; ageyevat@pt.bme.hu (T.A.); sibikini@pt.bme.hu (I.S.)

**Keywords:** ε-caprolactam, lauryllactam, in situ polymerization, anionic activated ring opening polymerization (AROP), copolymers, blends, nanocomposites, thermoplastic resin transfer molding (T-RTM), pultrusion, single-polymer composites

## Abstract

This paper presents a comprehensive overview of polymers and related (nano)composites produced via anionic ring opening polymerization (AROP) of lactams. It was aimed at surveying and showing the important research and development results achieved in this field mostly over the last two decades. This review covers the chemical background of the AROP of lactams, their homopolymers, copolymers, and in situ produced blends. The composites produced by AROP were grouped into nanocomposites, discontinuous fiber, continuous fiber, textile fabric, and self-reinforced composites. The manufacturing techniques were introduced and the most recent developments highlighted. Based on this state-of-art survey some future trends were deduced and as their “driving forces” novel and improved manufacturing techniques identified.

## 1. Introduction

Polyamides (PAs) belong to the engineering thermoplastics with a broad range of applications. Most of the PAs are synthesized in polycondensation using diamines and diacids or α-amino acids. The related step growth process results in versatile backbones, containing the recurring amide groups (–CO–NH–), which are either prone to crystallization or not. First patents on the preparation of polyamide-6,6 (PA-6,6) and polyamide-6 (PA-6) are dated back to 1937 and 1938, respectively [1]. However, PAs can be synthesized by polymerizing monomers with ring structures. This ring-opening polymerization (ROP) of cyclic monomers (lactams) is essentially a chain growth process. ε-caprolactam (CL; also referred to as hexano-6-lactam) and ω-laurolactam (lauryllactam) (LL) are cyclic monomer components of the amide group and five and eleven methylene (–CH_2_) groups, respectively. The commercially and technologically relevant CL and LL are the respective monomers of PA-6 and PA-12, respectively. The first description of the anionic polymerization of CL goes back to 1941 [1,2]. Since then a great number of works have addressed different aspects of the polymerization, properties modification, and processing of in situ produced PA-6 and PA-12. Nowadays, two topics are under the spotlight of interest for both academia and industry: production of nanocomposites and “traditional” composites via in situ anionic ring opening polymerization (AROP) of CL and LL.

Next, we survey the development in the last two decades following the grouping: homopolymers, copolymers, in situ produced blends, nanocomposites, and composites. The reader will find, however, some earlier references (especially with respect to polymerization mechanisms) which are still unsurpassable. The related section contains also information on processing and its modeling (when available). It is not out of place to underline that new processing techniques (electrospinning, additive manufacturing, AM) have given a new impetus to the AROP of lactams.

## 2. Homopolymers

### 2.1. Chemistry

The polymerization capability of cyclic monomers depends on both thermodynamic (relative stability of the monomer and the resulting linear polymer) and kinetic (initiation, propagation, termination reactions) factors. The presence of heteroatoms in the cyclic rings facilitates the nucleophilic or electrophilic attacks thereby accelerating the initiation and subsequent propagation reactions [3]. Therefore, the polymerization of lactams can be initiated by bases (anionic) and acids (cationic), as well. Note that initiation by water (hydrolytic polymerization) is the industrial polymerization technique of lactams, this is, however, not covered in this review. Cationic ROP is not straightforward because it yields low molecular weight (MW) products, even at low conversions.

Mechanisms of the anionic polymerization of lactams were deduced from studies performed mostly on CL, and summarized in excellent reviews already in the 1970s [4,5]. Few works addressed the AROP of LL which is the other technologically relevant lactam beside CL. Nevertheless, recent AROP works have also dealt with systems other than lactams [6]) and with lactams with smaller rings than CL (e.g., β-lactam [7,8]).

**Figure 1:**
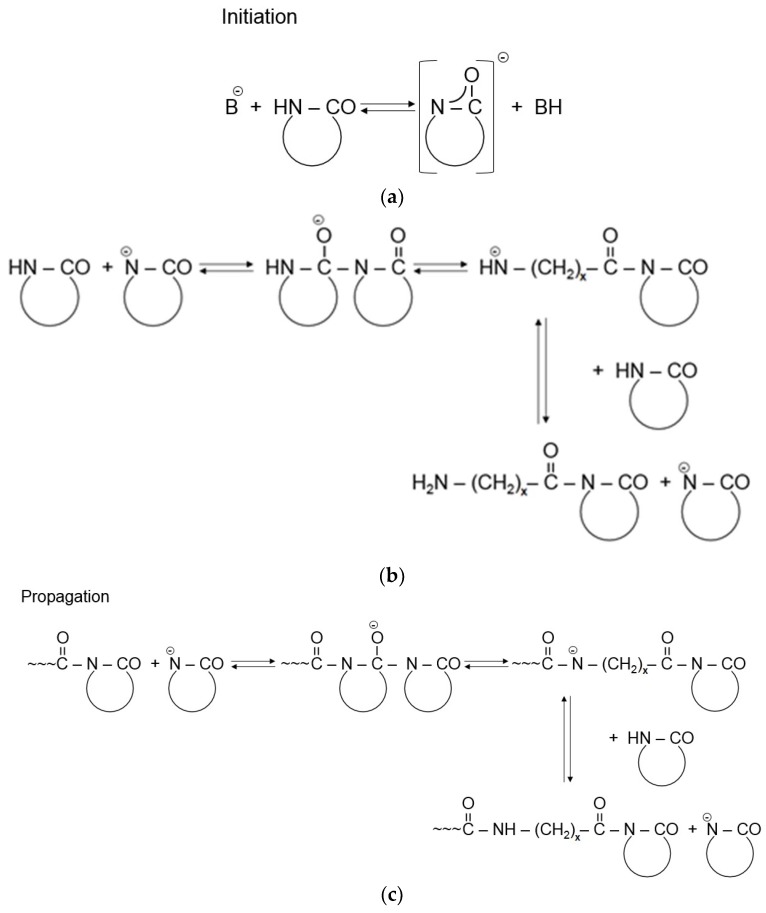
Initiation (**a**,**b**) and propagation (**c**) of the AROP of lactams, schematically. Note: x = 5 and 11 for CL and LL, respectively.

The AROP of lactams consists of the following steps [1,4,5]. Strong bases, forming free lactam anions, are the initiators (see Figure 1a). Initiation occurs by disproportionation including the ring opening of the lactam and final formation of a primary amine anion. Because this amine (aminic) anion is more basic than the initial lactam anion, through fast proton exchange a new lactam anion and ω-aminoacyllactam form. Propagation proceeds by repeated nucleophilic attack of the lactam anion and the endocyclic carbonyl group of the non-ionic growth center (see Figure 1c). Thus, the reaction involves the repeated acylation of the lactam anion (Figure 1b).

The development of the non-anionic growth center, i.e., *N*-acylated lactam, is the controlling step of the non-activated ROP of lactams. The polymerization of lactams initiated solely by lactam anions exhibits an autocatalytic character. Though the reaction scheme in Figure 1 suggests the onset of a living anionic polymerization [1], it is seldom referred to under this heading. The major reason for this is that besides the expected linear chain growth different cyclization and branching reactions [9,10] with formation of other groups than amides occur. These reactions may decrease the initiator’s content via formation of other active growth centers. Moreover, the newly generated groups (such as ketoamides) may undergo thermal decomposition associated with water release. Water is a strong inhibitor of AROP. However, initiators of the non-activated polymerization may show different sensitivities toward water [11].

Figure 1 already hints that the propagation rate of the anionic polymerization can be prominently enhanced by the introduction of in situ generation of compounds of the *N*-acyllactam structure (bearing an endocyclic carbonyl group) acting as growth centers (see Figure 1b). One needs to recall that this speed up of the polymerization is due to the fact that the nucleophilic attack of the lactam anion on the endocyclic carbonyl group of the activator is much faster than on the carbonyl group of the parent lactam.

The initiators, produced ex situ or in situ, are mostly salts (sodium) of lactams. The sodium salt of CL (sodium caprolactamate, NaCL), by whatever means produced, is the most widespread initiator. Nowadays, NaCL is commercially available under the trade names Bruggolen^®^ C10 (C10, Brüggemann Chemical, Heilbronn, Germany) and Addonyl^®^ CR CATALYZE and Addonyl^®^ Kat NL (Rhein Chemie of Lanxess, Cologne, Germany). Another commercially available initiator is the ε-caprolactam magnesium bromide (CLMgBr) offered as Nyrim^®^ C1 (C1) by Bruggeman Chemicals. CLMgBr likely participates in the lactamolytic mechanism, meaning metal complexing of the growth center and the lactam [1,4]. A further commercial initiator is sodium dicaprolactamato-bis-(2-methoxyethoxo)-aluminate (reaction product of sodium dicaprolactamato-bis-(2-methoxyethoxy)aluminum hydride (Synhydride) and CL), produced by Katchem (Prague, Czechia) under the trade name of Dilactamate^®^.

The activators are classified as direct or indirect ones. Direct activators exhibit an *N*-acyllactam structure, while compounds transformed to such structures in situ via suitable reactions and acting further as growth centers are termed indirect ones. By the selection of activators various end groups can be introduced into the polylactam. Needless to say this is accompanied by other side-reactions.

**Figure 2:**
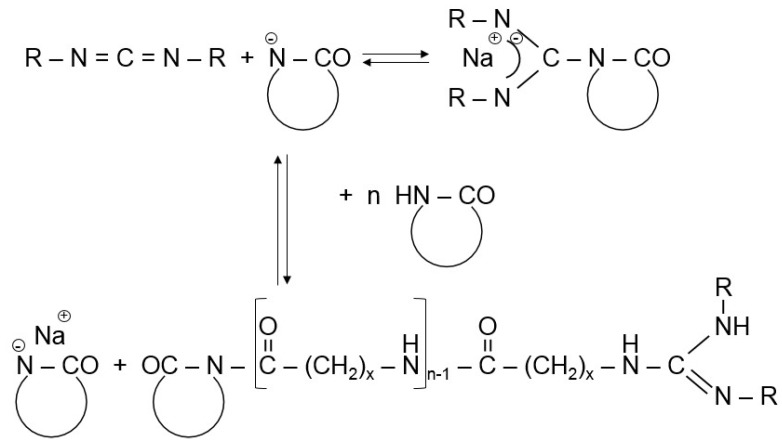
Polymerization of lactams using carbodiimide-type activator. For note see Figure 1.

As direct activator in the CL polymerization usually *N*-acetyl-ε-caprolactam was used along with NaCL or CLMgBr initiators [1,12]. Such systems were also used in model studies [13]. Other widely used direct or indirect activators are represented by *N*-carbamoyllactams. Bruggolen^®^ C20 (C20) for example contains hexamethylene-1,6-dicarbamoylcaprolactam as active species. The active substance in Addonyl^®^ ACTIVATE is a CL-blocked isocyanate. *N*-carbamoyllactam precursor polyisocyanates are offered by Rhein-Chemie (Lanxess) under the trade names Addonyl^®^ 8101 and Addonyl^®^ TT. Polycarbodiimide-based activators are also commercially available (Addonyl^®^ P). The growth center from carbodiimide and alkali lactamate has a guanidine structure—see Figure 2. Because the guanidine anion is stabilized by resonance, it undergoes fast proton transfer followed by rapid chain propagation—see scheme in Figure 2 [14]. This activator was the essential part of the initiator/activator package, developed by EMS Chemie (Domat, Switzerland), for the anionic LL polymerization (see later).

The search for efficient activators for linear and branched polylactams is still ongoing. Russo et al. [15] mixed a 3-(triethoxysilyl)propylisocyanate blocked with CL, which proved to be a fast activator. Multifunctional activators, yielding branched polyamides, were initially prepared by blocking tri- or multifunctional polyisocyanates with CL [16]. Note that blocking of isocyanates is a straightforward method to reduce the eventual toxicity and water sensitivity of polyisocyanates. In a recent work Zhu et al. [17] used *N*,*N*′,*N*′′-trimesoyltricaprolactam as multifunctional activator. Mateva and Dencheva [18] synthesized organophosphorus lactam derivatives as potential activator for the AROP of CL. Their efforts addressed also the simultaneous improvement of fire resistance and thermos-oxidative stability of PA-6.

The most noticeable recent development in the field of initiators can be assigned to Buchmeiser et al. [19]. The cited authors synthesized a protected *N*-heterocyclic carbene compound which acted as latent pre-initiator in the polymerization of lactams (CL, LL). The carbon dioxide or metal salt protected carbenes—after cleavage of the protected groups—are strongly basic and thus initiate lactam anion formation. Unfortunately, no information was released about the effects of carbenes in the presence of activators.

Attempts were also made to influence the progress of anionic lactam polymerization through additives which may cause complexation with the counter-ion or influence the anion formation and anion stabilization. For such purposes crown compounds [20] and ionic liquids [21] have been tried.

For further information on initiators, activators, and mechanisms of the AROP of lactams the interested reader is advised to consider the excellent books, monographs and reviews, indicated in Refs. [1,3,4,5].

### 2.2. Properties

The anionic polymerization of lactams can be performed in two temperature ranges: below or above the melting temperature (*T*_m_) of the resulting polylactams. Note that *T*_m_ of PA-6 and PA-12 lie in the temperature ranges 210–225 °C and 180–190 °C, respectively. The polymerization temperature affects the conversion, MW, crystallinity and thus also the thermomechanical performance of the product. Polymerization above the *T*_m_ may be necessary due to the actual processing or when the polymer itself is the final target. It should be born in mind however, that polymerization above *T*_m_ may result in relatively high cyclic monomer concentration. Its amount may be as high as 8–10 wt % in PA-6, whereas it is much lower in PA-12 (~2 wt %). By contrast to PA-12 (tolerating this macrocyclic content), the residual monomer in PA-6 should be removed by extraction [1].

The conversion of the cyclic monomer to linear polymer can be followed by different techniques. For quantification of the residual CL extraction (water, methanol), vacuum drying, and thermogravimetric analysis (TGA) may be used [22]. The two latter techniques make use of the fact that CL easily evaporates (its boiling temperature is below 270 °C which is below the thermal degradation of PA-6). A more advanced method for monomer determination is represented by size exclusion chromatography (SEC, also termed as gel permeation chromatography, GPC). A further advantage of SEC is that it informs us about the MW and its distribution. Suitable SEC eluents for PA-6 are fluorinated alcohols [23], whereas for PA-12 benzylic alcohol may also be used [24]. For MW determination solution viscosimetry is the traditional tool. The (Landau-Kuhn)-Mark-Houwink-(Sakurada)—the names in brackets are often not mentioned—equation correlates the intrinsic viscosity with the MW (viscosity—average value, *M*_v_). The usual solvents are sulfuric acid (various concentrations), formic acid, and cresol compounds.

The MW of PA-6 and PA-12 may reach 70–100 kDa [24] though the usual MW range is 20–30 kDa. Luisier et al. [25] indicated 26–41 kDa range for commercialized PAs. These authors synthesized PA-12 using the “one-component” initiator/activator (sodium lactamate/cycloaliphatic monocarbodiimide) of EMS Chemie and found the MW between 20 and 60 kDa depending on the amount of the liquid initiator/activator system.

Polymerization below *T*_m_ is favored as it yields high conversion (degree of conversion, DOC = 96%–99%) and high crystallinity (40%–50%). Note that PAs crystallized from the melt exhibit lower crystallinity values (30%–40%) [1]. A major benefit of polymerization below *T*_m_ is, however, that it is accompanied by fast solidification owing to crystallization.

Crystallization takes place after or simultaneously with the polymerization. So, crystallization occurs in an undercooled melt. This feature is very similar to another technologically relevant cyclic oligomer, namely cyclic butylene terephthalate (CBT), which polymerizes into polybutylene terephthalate (PBT) via ROP [26]. The occurrence of polymerization and crystallization (whether subsequent or simultaneous) was a topic of intensive research. Karger-Kocsis and Kiss [27] concluded, based on differential scanning calorimetry (DSC) results, that polymerization and crystallization are superimposed processes in the anionic activated polymerization of CL. This was confirmed in a recent paper [28]. The cited authors also demonstrated that the polymerization/crystallization during heating and crystallization of the PA-6 formed upon cooling may be highly different as a function of the activator composition. The detailed DSC study of Vicard et al. [29] shed light on the course of monomer polymerization and simultaneous crystallization of the growing chains. It was found that at high temperature (isothermal scan) or high heating rate (dynamic DSC scan) polymerization precedes the crystallization because of the small extent of supercooling (undercooling). Under a given condition those processes may be fully decoupled which again is very similar to that of CBT polymerization [30]. Based on the complete separation of the polymerization and crystallization the total enthalpy of polymerization could be determined (~123 J/g) [29]. It is noteworthy, that by enlarging the ring structure the reaction enthalpy is reduced. Luisier et al. [14] reported 53 J/g as enthalpy value for the activated anionic polymerization of LL.

Both PA-6 and PA-12 are semicrystalline polymers. Each of them has two basic crystalline structures such as α monoclinic and γ (pseudo) hexagonal. Their appearance depends not only on the synthesis and processing conditions but also on other factors, like additives. It is shown later that incorporation of nanofillers may cause a change from the more stable α to the less stable γ-phase.

As underlined above the rate of polymerization and crystallization strongly depends on the temperature (below or above *T*_m_, isothermal or adiabatic conditions), as well as on type/amount of the initiator/activator. A large body of works addressed this issue aiming to derive proper kinetic models. The related experimental work was often supported by powerful statistical analysis [31]. Russo et al. [32] compared different kinetics models and claimed that that of Kamal and Sourour [33] fits best for the activated anionic polymerization of CL. To adapt this, an autocatalytic model (which is widely used to describe the curing of various thermosets [34]) was proposed by Teuwen et al. [35]. Moreover, the cited author recommended using the isothermal crystallization model for the crystallization of the PA-6 formed during the anionic polymerization of CL. The prediction of polymerization/crystallization in the AROP of lactams is essential for process modelling. The polymerization kinetic approach of Malkin [36] was implemented by Nagy et al. [37] in a fluid dynamics code to estimate the flow of the polymerizing CL during mold filling.

The fact that the polymerization of CL can be adequately described by the Kamal-Sourour equation, developed for thermoset curing, already hints at some similarities between the in situ polymerization and curing of thermoset resins. Basically in both cases the viscosity (chemorheology) and conversion change (increase) as a function of time and temperature. By contrast to thermosets, however, no gelling, vitrification and cross-linking take place during AROP of lactams yielding linear or only slightly branched PAs.

For thermosets the above changes are summarized in conversion-temperature-transformation (CTT) and time-temperature-transformation (TTT) diagrams. These diagrams are of tutoring character to understand the curing, and even more importantly, to optimize the curing process [38]. The knowledge of the time-temperature route of the AROP is a key factor to set up various industrial processes. Pioneering work in this direction was done by Luisier et al. [14]. The cited authors constructed a TTT diagram for the anionic polymerization of LL. The input parameter (conversion, crystallinity) was derived from isothermal and dynamic DSC scans. Unlike thermosets for which the *T* parameter (*Y*-axis) is the glass transition temperature (*T*_g_), here this role belongs to the polymerization temperature. The TTT diagram indicated the change in the DOC and onset of eventual crystallization at polymerization temperatures selected both below and above the *T*_m_ of PA-12.

**Figure 3:**
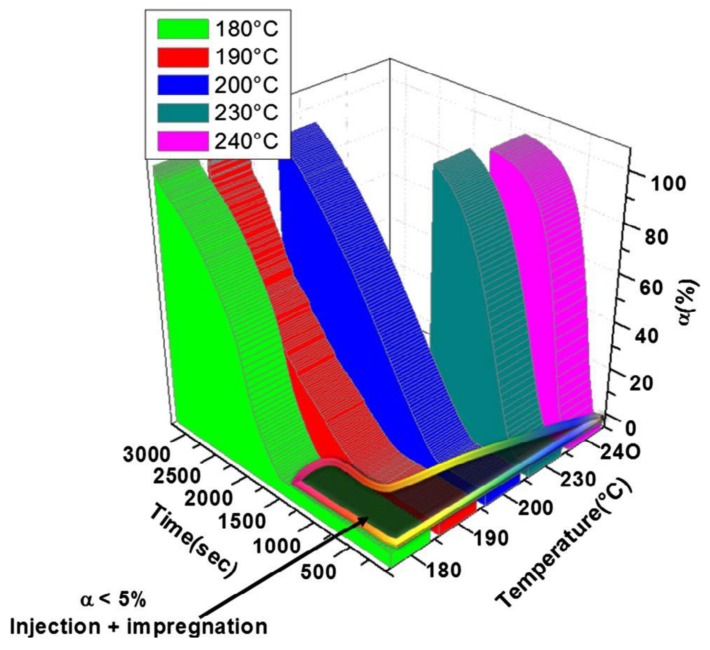
TTT isothermal reaction diagram for the anionic activated CL polymerization. Notes: α means the conversion determined by FTIR. The composition contained NaCL initiator (Bruggolen C10) and diisocyanate-based activator (Bruggolen C20P) in 4 parts per hundred-part CL (phr) each ([39] reproduced with the permission of Elsevier).

The recent paper of Maazouz et al. [39] puts emphasis on the chemorheology, i.e., viscosity change as a function of both polymerization temperature and time, when creating a TTT diagram for the anionic activated (“fast”) polymerization of CL. The authors followed the polymerization with different experimental techniques to get information on the conversion (Fourier-transform infrared spectroscopy, FTIR) and viscosity (parallel plate configuration combined with in situ FTIR). Moreover, an attempt was made to use dielectric measurement for assessing the course of polymerization. This technique, that was already tried for the polymerization of CBT [38], is sensitive to changes in the ionic conductivity, and may serve for in-line quality assurance of production. Dielectric technique cannot, however, differentiate well between polymerization and crystallization induced changes. To detect the latter a more reliable technique may be the assessment of crystallization induced shrinkage using Fiber Bragg monitoring—which again has already been tried for the in situ polymerization of CBT [40]. The TTT diagram (see Figure 3) of Maazouz et al. [39] is of great practical relevance because it informs us about the presence of a viscosity range lower than 1 Pa∙s. Note that 1 Pa∙s is generally considered as the maximum threshold of all resins used in resin-transfer molding (RTM) or similar operations.

### 2.3. Manufacturing

PA-6 and PA-12 homopolymer products in forms of plaques, pipes, rods, and various half-fabricates for further machining, are produced by casting (see the related scheme in Section 6). Casting is likely the oldest processing of anionically polymerized lactams. Even in recent reports the term “monomer cast nylon” or the like may appear (see the titles in the reference list). Cast products are large parts which either cannot be realized or are not-economical for injection or compression molding. For some specific tribological applications (bearing, gear) cast PAs are still unavoidable.

Nowadays, however casting has received a powerful competitor, viz. additive manufacturing (AM). Pioneering activities on the application of anionic polymerization of lactams for AM versions started in the 2010s. Khodabakhshi et al. [41,42] used traditional initiators (NaCL, CLMgBr) and activator (*N*-acetylcaprolactam) in “drop on drop” and DSC tests to determine the optimum formulation and conditions for ink-jetting. The solidification half-time, linked with the productivity of this AM method, could be reduced to below one minute. The authors compared the characteristics of their ink-jetted PA-6 with those of cast PA-6s. The conversion, *M*_v_ and crystallinity of the ink-jetted PA-6 were 95%–96%, 41–50 kDa and 38%–40%, whereas those of the cast PA-6s exhibited 94%, 45–52 kDa and 43%–44%, respectively [42]. It can be predicted that the next step in AM via the anionic polymerization of lactams will be their modification, especially with nanofillers [43].

**Figure 4:**
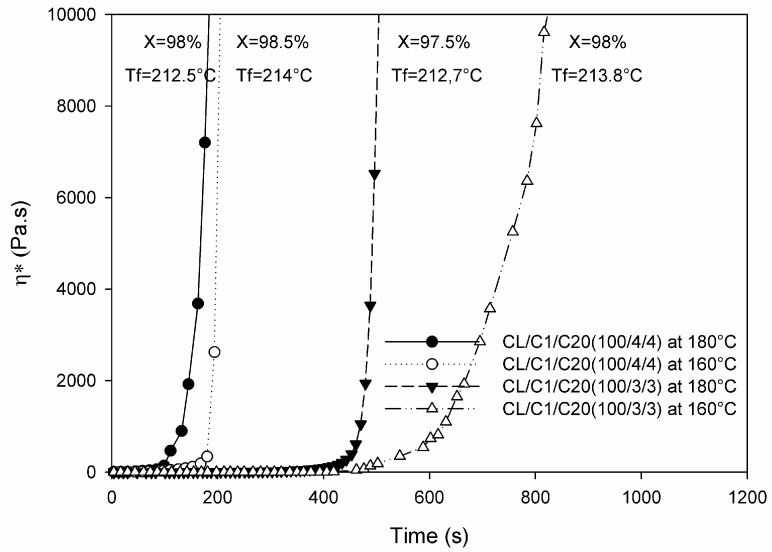
Change in the complex viscosity as a function of time, temperature, and initiator/activator formulation. Designation: *X*—conversion, *T*_f_—fusion temperature, CL—ε-caprolactam, C1—Bruggolen C1 (CLMgBr in 1.4 mol/kg concentration in CL), C10—Bruggolen C10 (NaCL in 1 mol/kg concentration in CL), C20—Bruggolen C20 (hexamethylene-1,6-dicarbonoyl caprolactam; 2 mol/kg concentration in CL) ([46] reproduced with permission from BME PT).

The other “old” processing techniques making use of the AROP of lactams are centrifugal and rotational molding (see the related schemes in Section 6). Products of centrifugal molding are rods, tubes, and pipes while large hollow bodies are produced by “reactive” rotational molding [44]. In these processes polymerization and shaping occur at the same time (net-shape processing). It is noteworthy that the related process simulations are of practical interest [44]. In these operations the polymerization takes place below the *T*_m_ of the final product thereby reducing the cycle time (fast demolding owing to solidification). Rusu et al. [45] studied the polymerization of CL and CL + LL in centrifugal molding at *T* = 160 °C using CLMgBr initiator and mono- and bifunctional activators (*N*-benzoyl-ε-caprolactam and *N*,*N*′-isophtaloyl-bis caprolactam, respectively). The maximum conversion reached was ~98%. MW characteristics, also deduced from GPC, changed prominently as a function of the activator’s amount and type. The polydispersity was ~4 and between 6 and 11 for the mono- and bifunctional activators, respectively, confirming that branching, crosslinking was caused by the bifunctional activator. Barhoumi et al. [46] investigated the anionic polymerization of CL during rotational molding. As initiators NaCL and CLMgBr were selected, whereas as activator the bifunctional hexamethylene-1,6-dicarbamoylcaprolactam (products of Brüggemann Chemicals) was selected and the polymerization performed at different temperatures below the *T*_m_ of PA-6. The induction time of the starting polymerization depended on the temperature, type and amount of the initiator/activator formulation—see Figure 4.

To support the reactive rotational molding of in situ polymerized PA-6 the authors summarized the chemorheological results (isothermally heated) in TTT diagrams. Considering the fact that also for rotational molding the ideal viscosity should be less than 1 Pa·s, the processing window (time available at a given temperature to reach this threshold) could be well defined [46]. In this study features of classical (PA-6 powder/flake) and reactive rotomolding (anionic polymerization of CL) were compared. Table 1 clearly shows the benefits of reactive rotational molding over traditional ones [46].

Considering the fact that the anionic polymerization of lactams is much faster than the hydrolytic one, several attempts were made to synthesize PAs through reactive extrusion. Credit in this field should be given to the groups of Michaeli [47] and White [48,49]. The related works covered not only homopolymers but also other modifications.

Wu et al. [50] tried to summarize the achievements in reactive extrusion of lactams via numerical simulation. Interestingly, in situ foaming of anionically polymerized lactams was not yet a topic of academic research. The patent literature [51] suggests, however, that this is a feasible option. When the polymerization is performed below *T*_m_ then the decomposition temperature of chemical foaming agents should be carefully selected.

A peculiar feature of the anionic polymerization of lactams is that it represents the only suitable method to produce PAs in powder form [1]. To get powder the polymerization proceeds in suitable organic liquids acting as precipitants. Oily polyisobutylenes proved to be very suitable dispersing fluids for the micronscale PA powders [52]. Crespy and Landfester [53] developed a mini-emulsion process yielding PA-6 with *M*_v_ of ~35 kDa. The major advantage of this process was that PA-6 particles in nanoscale (15–30 nm) could be received. This was a big achievement as the traditional techniques resulted in particles ranging from a few to hundreds of microns [1]. Note that the applications of PA-6 powders, especially the nanoscaled ones, are manifold (medical, sensoric, environmental) [54].

## 3. Copolymers

Extensive research was dedicated to the modification of anionically polymerized PA-6 and PA-12 through various copolymerization strategies. The property modifications mostly aimed at reducing the crystallinity and melting temperature, enhancing the ductility and toughness, as well as improving the thermal and hydrothermal resistances. Next, we give a brief survey on various copolymerization techniques classified according to the comonomer pairs or molecular build-up.

### 3.1. Chemical and Structural Aspects

#### 3.1.1. Lactam-Lactam Copolymers

Anionic copolymerization is focused on the combination of CL and LL as industrially available lactams. The copolymerization of CL with LL is generally performed in the temperature range 130–180 °C, i.e., below *T*_m_ of PA-6 and close to the *T*_m_ of PA-12. The related formulations usually contain more CL than LL because CL is more reactive that LL. Based on the topic-related summary of Roda [1], the activated anionic polymerization with NaCL initiator yielded random copolymers. These random copolymers exhibit low *T*_m_ (minimum value ~135 °C) and reduced crystallinity compared to PA-6. In contrast, the copolymers synthesized with CLMgBr initiator displayed two distinct *T*_m_s in the ranges *T* = 130–140 °C and *T* = 200–220 °C, respectively, when the actual CL content was between 30 and 70 mol %.

Kinetic investigations confirmed that in the initial stage of polymerization PA-6 homopolymer forms, while random chain, consisting of CL and LL, appears in a later stage [1,55]. Ricco et al. [56] studied the activated anionic copolymerization of CL and LL (the latter varied up to 33.3 mol %) at 155 °C. As initiator sodium lactams (NaCL or Na(CL + LL)), whereas as activator hexamethylene dicarbamoylcaprolactam was selected. This work was focused on changes in the thermal and structural properties thereby considering the products of side reactions. With increasing LL content *T*_m_ was reduced but its change as a function of composition did not obey Flory’s prediction. De-oligomerization by extraction affected *T*_g_, *T*_m_ and the crystallinity of the samples. Wide and small angle X-ray scattering (WAXS and SAXS, respectively) results suggested that LL incorporation leads to reduced crystallinity and less perfect crystals.

Nauman et al. [57] copolymerized LL with CL at *T* = 180 °C using no activator but a thermally latent, protected *N*-heterocyclic carbene as initiator. The authors reported on the formation of a gradient copolymer because CL was incorporated into the polymer backbone preferentially in the starting phase of polymerization. Rusu et al. [45] studied the activated anionic copolymerization of CL and LL in centrifugal molding using ethyl magnesium bromide (converted in situ into CLMgBr) initiator and *N*,*N*′- isophthaloyl-bis-ε-caprolactam activator at 160 °C. The LL amount in the feed was varied between 0 and 50 wt %. It was found that the conversion, *M*_v_, and degree of crystallinity were all reduced with increasing LL content. Parallel to that, however, the flexural modulus and water uptake decreased while the Izod impact strength increased. Vygodskii et al. [58] copolymerized CL with LL using CLMgBr initiator and *N-*acetyl-ε-caprolactam and an aromatic polyimide as activator at *T* = 150–170 °C. The conversion (<75%) depended on the actual CL/LL ratio in the feed and on the initiator/activator selection including their relative amounts. The above mentioned results underline that the copolymerization of lactams is a very complex process. The relative amount of the comonomer built-in and their position along the polymer chain depend on several factors (reactivity of each activated monomer, acylation of the lactam anions, possible side reactions, crystallization reactions etc.).

#### 3.1.2. Lactam-Lactone Copolymers

Lactones can be treated as activators of the anionic polymerization of lactams, at least until their amount remains below 5 mol % [1]. In this case the initiation step is the acylation of lactone by the lactam anion [1,59]. At higher concentrations, lactone may work as activator and comonomer at the same time. The acylated lactone growth center (alkoxide anion) prefers the polymerization of lactone resulting in a polyester chain segment because the incorporation of lactams is much slower. Therefore, one would expect the formation of a block copolymer. Against this expectation, however, random copolymers appear due to extensive transacylation and transamidation (“trans” reactions) [1]. Nevertheless, lactam–lactone block copolymers can be synthesized when the copolymerization is kinetically controlled. A suitable controlling tool in the reactive extrusion may be the separate, sequential feeding of the lactam (containing both the initiator and activator) and the lactone (usually ε-caprolactone) [60,61]. The block length in this poly(amid-block-ester) could be controlled by the feeding ratio of each monomer during extrusion. Microwave irradiation was also applied to synthesize poly(ε-caprolactam-*co*-ε-caprolactone) directly via the AROP of the corresponding cyclic monomers. Fang et al. [62] reported that both monomers absorb microwave in the range of 0.4 to 3 GHz. In this study only initiator was used, namely LiCL produced in situ from lithium-tri-tert-butoxyaluminohydride. The conversion of the poly(amide-*co*-esters) was up to 70%. The structure of these copolymers was random and their *T*_g_ could be well predicted by the Fox equation. Experimental variables of the copolymerization were the initiator’s level, reaction time, and temperature. Although ε-caprolactone is the preferred lactone component of the copolymerization with lactams, other lactones containing 4–7 C atoms may also be used [63].

#### 3.1.3. Block Copolymers

The preparation of copolymers (di-, triblock) with blocks composed of lactam or non-lactam chains requires the incorporation of suitable prepolymers in the molten, anionically polymerizable lactam. Such prepolymers should be soluble in the lactam melt and, in addition, bear reactive end groups, such as hydroxyl (–OH) and amine (–NH_2_). The most important step of the block copolymerization is to convert the end groups of the polymer to *N*-acyllactam or *N*-carbamoyllactam moieties. Afterward, they function as non-ionic growth centers, i.e., macroactivators, in the subsequent anionic polymerization of CL or LL. The converting precursors are polyisocyanates (*N*-carbamoyllactam) or bis-acyllactams undergoing exchange reactions (alcoholysis, aminolysis) [1]. Poly (ε-caprolactam-block-polyether) copolymers were produced, and the related components offered commercially by Monsanto, in 1982 (Nyrim^®^ technology). The related block copolymers are often referred to as nylon block copolymers (NBC) and another widely used name is nylon reaction injection molded (RIM). Though Monsanto terminated its Nyrim^®^ activity in 1985, it was overtaken by DSM in 1986 [64], and now it is owned by Brüggemann Chemicals and commercial again.

**Figure 5:**
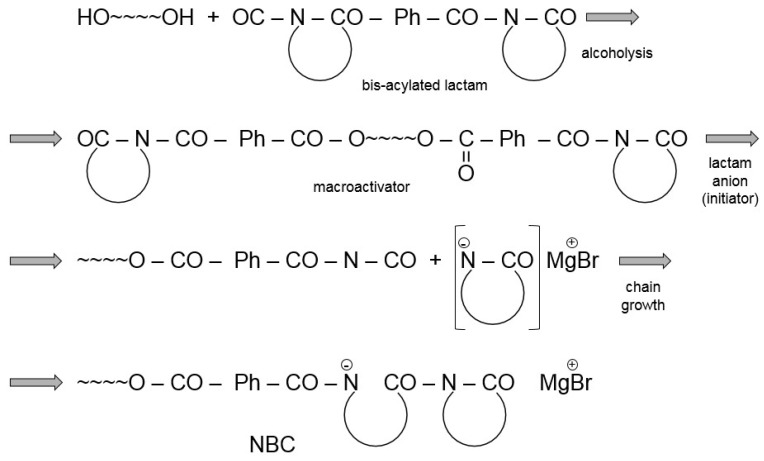
Preparation of poly(amide-block-ether) or poly(amide-block-ester) according to the Nyrim^®^ technology scheme.

This nylon reaction injection molded (Nylon RIM, NBC) block copolymer is obtained in the activated anionic polymerization of CL in the presence of polyols (polyether- or polyester-types) and bis-acyl derivatives of CL [65]. The initiators are usually NaCL or Grignard reagents (e.g., alkoxy-magnesium-bromide yielding CLMgBr in situ. Although the water uptake of NBC is lower than that of PA-6, efforts were made to reduce it further and to increase its resistance to hydrothermal aging. Improvements in this relation were achieved by incorporating phenolic resin [66,67]. According to the related patent [68] the MW of the polyol is higher than 2 kDa. The reaction scheme of NBC preparation is given in Figure 5.

In the NBC formulations mostly polyether and polyester appeared as block segments. However, their end groups were not always hydroxyls but amines. This change is well understandable considering the fact that such amines (e.g., polyetherdiamines) are widely used as hardeners for epoxy resins. The amine end groups of such polyethers were transformed to the necessary macroactivator via aminolysis with carbamoyl compounds before starting with the anionic polymerization of CL. The resulting block copolymers exhibited high MW and polydispersity which were traced to the onset of Claisen-type condensation reaction. Amines were the functional groups of the growth center generating polyether compounds of the group of Ye [69,70]. This group synthesized, however, the macroactivator with carbamoyl moieties by reacting the amine end groups with polyisocyanates. The block copolymers showed excellent toughness and even antistatic properties. Toughness improvement was the target of the work of Kim et al. [71], who used different polyetherdiols and polyetherdiamines as precursors for the blocks, and mono- and dicarbamoyl caprolactam compounds to transform them for growth centers (macroactivators) via exchange reactions. It was found that differences in the block forming polymers and related macroctivators strongly affect the conversion, crystal structure and thermomechanical properties of the outcoming block copolymers [71]. The majority of the block forming prepolymers were polyether-types. Polyether-type diols, such as polycaprolactone (PCL) were also used to prepare poly(amide-block-ester) copolymers. Kim and White [72] end capped PCL with di-isocyanates to generate the macroactivator supporting the anionic polymerization of LL.

As block segments other polymers than polyethers and polyesters may be inserted. Sobotik et al. [73] applied α,ω-dihydroxy polybutadiene that was transformed to macroactivator by reacting with various diisocyanates. The final product was poly(amide-block-butadiene). The block (soft segment) may show rubbery characteristics. Note that liquid butadiene rubbers with suitable functionality, such as amine, were developed for the toughening of epoxies and they have been tried as prepolymers in the anionic polymerization of lactams, as well.

Rached et al. [74] produced a triblock copolymer (PA-12-block-polydimethylsiloxane (PDMS)-block PA-12) containing siloxane block segment. PDMS is water repellant and has a very low *T*_g_. Therefore, its incorporation seems to be the right selection for toughening and to reduce water sensitivity. In this work the silanol end groups of linear PDMS were reacted with diisocyanate that was converted into the macroactivator, viz. α, ω-dicarbamoyloxy CL-PDMS. The latter activated the anionic polymerization of LL. The MW of the soft block PDMS was varied between 3 and 24 kDa. The MW of the polymerized hard PA-12 end blocks were in the range of 10–250 kDa. The authors stated that this block copolymer may work as compatibilizer for PA/PDMS blends.

There are some further possibilities to create block copolymers. Phenylester groups may also work as latent activators. Phenylester groups were generated on a polyimide (PI) backbone by end-capping with phenyl-4-aminobenzoate in *N*-methyl-2-pyrrolidinone solvent [75]. After precipitation and drying this activated the anionic polymerization of CL at *T* = 120 °C. The thermal stability, moisture resistance, and impact strength of the PA-6-PI-PA-6 block (and grafted—see later) copolymers were markedly enhanced [75].

#### 3.1.4. Graft Copolymers

Copolymers are rarely formed by interfacial reactions. The anionic polymerization of lactams may open up a bright horizon, however, in this direction as outlined below. To graft PA onto a polymer, the latter has to be transformed into a macroactivator through suitable chemistry. The other aspect to be considered is whether this polymeric macroactivator is soluble only in a common solvent with lactams or in the melt of the latter (which is strongly preferred). Hu et al. [76,77] developed a method to graft PA-6 into PP (PP-*g*-PA-6). This was done by functionalizing the PP chain with isocyanate (–NCO) groups. This overtook the role of macroactivator when the isocyanate groups reacted with the CL anion (PP-carbamoyl CL). The selected polymerization temperature was in the range of 200–220 °C, i.e., above the *T*_m_ of PP. The authors emphasized that this PP-*g*-PA-6 could be produced by reactive extrusion and may be a good compatibilizer for PP/PA-6 blends. Recall that the target with copolymerization was to improve some disadvantageous properties of PA (low *T*_g_, low toughness, high water uptake, moderate thermooxidative stability). To reduce the moisture sensitivity of PA-6 it is often blended with polyolefins, such as PP. Zhang et al. [78] grafted PA-6 onto a polystyrene (PS) chain by a similar approach. The basic difference compared to that of Hu [76,77] was that PS itself was a copolymer containing styrene and an isocyanate group bearing styrene derivative. The strategy was similar to the above one in the work of Liu [79] who synthesized a copolymer composed of styrene and an allyl monomer having a carbamated caprolactam moiety. PA-6 chains were grafted on this styrenic macroactivator via anionic polymerization of CL at *T* = 170 °C.

The above examples underline that copolymerization is a versatile route to incorporate a monomer with suitable groups (i.e., *N*-acetyl, *N*-carbamoyl) or with functional groups which react with the lactams thereby generating the macroactivator (growth center). A further example in this direction can be taken from the work of Xu et al. [80]. These authors copolymerized styrene with *N*-phenyl- and *N*-(4-hydroxyphenyl)- maleimides yielding poly(styrene-*co*-maleimide) copolymers. The phenolic –OH groups were afterward reacted with one –NCO group of a diisocyanate. The other –NCO group was reacted with the lactam resulting in the macroactivator for the anionic polymerization of CL. The poly(styrene-*co*-maleimide) with *N*-carbamoyl moities was synthesized in solvent, but the CL grafting was done in the melt at *T* = 170 °C. Grafting enhanced the *T*_g_ and reduced the crystallinity of the grafted PA-6 compared to PA-6, whereas their crystal structure was identical (α-form). Macroactivation with pendant phenylester groups may work for CL polymerization as shown by Pay [75]. The conversion of CL with PhMgBr initiator mat *T* = 120 °C after 10 min was up to 96%.

#### 3.1.5. Specialty Copolymers

Latent CO_2_-protected *N*-heterocycle carbene based initiators were also applied for the anionic copolymerization of CL with LL [81]. Recall that this is a single initiator that is incorporated into the lactam or lactam blends directly. The *M*_v_ values of the copolymers were at about 30 kDa along with a polydispersity close to 2. On the other hand, the oligomer content was very high (~40%). Bouchekif et al. [82] investigated the anionic polymerization of CL in the presence of urea- and amide-based bis-monomer yielding copolymers and cross-linked PA6 versions. Tunc et al. [83] prepared aliphatic PA bearing fluorinated groups via AROP of CL with α-perfluorobutyrylamido-ε-caprolactam. This copolymer exhibited enhanced *T*_g_, higher resistance to thermo-oxidative degradation but reduced *T*_m_ compared to PA-6. The presence of the fluorinated groups rendered this copolymer hydrophobic.

Volkova et al. [84] demonstrated that given PIs, without any functionalization, may be excellent activators of the anionic polymerization of CL. It was quoted that through selection of PI (chemical build-up, amounts) the mechanical and tribological properties of the resulting copolymers can be controlled. The work of Hou et al. [85] addressed the blending of thermoplastic polyurethane (TPU) via in situ AROP of CL. The reason why this approach is reported here and not in the following blend section is, that the authors proposed the onset of a copolymerization reaction. According to their polymerization pathway TPU undergoes thermal dissociation in the alkaline CL melt. Thermal dissociation yields –NCO groups which reacting with CL generate macroactivators. The authors found that with increasing content of TPU (up to 10 phr) the stiffness and strength dropped and the notched impact strength prominently increased.

Bakkali-Hassani et al. [86] developed a novel route for copolymerization thereby combining the AROP of CL with condensation reaction using esters (ethyl-4-butylaminobenzoate). The authors varied the content of the aromatic units in this aliphatic-*N*-alkyl aromatic copolymer between 3 and 27 mol %.

### 3.2. Manufacturing

The preparation method of the copolymers is identical—both in laboratory and industrial scales—with those repeated for PA homopolymers. Copolyamides were produced by casting [87,88], melt spinning [87], reactive extrusion [89], centrifugal [45] and rotational molding [90]. Several reports [89,91] have dealt with the effects of processing parameters on the thermomechanical behavior of the copolyamides. Literature data support the fact that reactive extrusion may be the most efficient industrial technology when granulated, pelletized copolyamides for further processing, are the target materials.

## 4. Blends

Blending of immiscible polymers envisages creation of blends with superior properties to those of the blend components. Preparation of the blends depends prominently on their morphology and adhesion between the phases of the blend. The morphology development during classical melt blending of “premade” polymers depends on many material- and processing-related parameters, including the actual viscosity ratio of the blend’s components. To improve the adhesion between the blend, constituent compatibilizers, compatible with both phases, are used. Blending of polymers combined with the in situ AROP of lactams is a very promising route, because the morphology development may be kinetically controlled and copolymerization, supporting the formation of a strong interphase, may be triggered at the same time. With respect to morphology development attention should be paid to some similarities with the toughening of thermosets. Tougheners, reactive or non-reactive with the actual thermosetting resin, are first dissolved in the resin or its hardener. The final morphology is given by reaction-induced phase separation (RISP) since the solubility of the toughening agent becomes less and less with the advanced curing of the resin [92].

When the modifier has co-reactive groups with those of the resin the separated phase may well be coupled to the matrix. Blending of polymers by simultaneous anionic polymerization of lactams is similar. The polymer modifier, as blend component, is dissolved in the lactam melt prior to the start of the anionic activated ROP of the latter. Even the compatibilization can be achieved in one-shot making use of the various copolymerization options introduced above. As a consequence, blending may involve copolymerization and vice versa. Therefore, it is not always a simple task to differentiate between blending and copolymerization when AROP of lactams is adapted. The distinction is, however, simple when the compatibilizer is introduced separately, being a previously synthesized polymer. The blend components in the in situ anionic polymerization of lactams are almost exclusively thermoplastics, though trials were made also with the combination of thermosets, such as epoxy resin [93]. A large body of work was done with the in situ blending of lactams with many different thermoplastics. Some of them were even produced in situ in the lactam melt before the AROP of the latter, others were premade and usually dissolved in the molten lactam. The related achievements are briefly summarized below.

Fang and Yang [94] investigated the structure-property relationship of low density polyethylene (LDPE) and in situ polymerized PA-6 in the presence and absence of a compatibilizer. This polymer compatibilizer was a maleic anhydride (MA) grafted LDPE (LDPE-*g*-MA). In situ compatibilization was achieved by grafting CL onto the LDPE-*g-*MA backbone via opening of the anhydride ring with NaCL. It is noteworthy, that the MA content of such grafted (maleated) polyolefins is usually about 1%. The measured CL conversion was ~92%. The dispersed particle size of the PA-6 in the LDPE matrix (content in the blend 70%) was reduced from ~2.5 to 1.5 μm through the in situ compatibilization. Du and Yang [95] adapted the same concept but using LDPE-*g*-MA in the AROP of LL. In this study the PA-12/HDPE mass ratios were varied between 100/0–60/40. The *M*_v_ values of PA-12 in the blends were between 11 and 22 kDa as a function of blend preparation (batch mixing or reactive extrusion). With increasing LDPE-*g*-MA content (up to 30 wt % LDPE-*g*-MA formed the dispersed phase in the PA-12 matrix) the stiffness and strength of the blends decreased while the toughness strongly increased.

Xu and Ye [96] prepared PA-6/polyethylene oxide (incorporating up to 7 wt %) blends through anionic polymerization of CL (called as “monomer casting nylon-6–based blend”). The crystallization of both the high MW polyethylene oxide and the PA-6 formed was influenced mutually. The particles of polyethylene oxide, well dispersed in the PA-6 matrix, supported the toughening via the enhanced ability of PA-6 for crazing and shear yielding.

The work of Teng et al. [97] was devoted to polypropylene (PP)/PA-6 blends in the composition range of 60/40 to 40/60. As compatibilizer, PP-*g*-MA was selected. For its action, imide formation between the MA group and primary amine of the PA-6 was supposed which is likely incorrect. Blends were produced in AROP of CL at *T* = 230 °C. The domain size of PA-6, dispersed in the PP matrix, was smaller than obtained in classical melt blending of PP and premade PA-6. The PA-6 particle size was reduced from >2 μm to <0.5 μm in the presence of the compatibilizing PP-*g*-MA.

Many works were performed to produce PA/styrenic blends via in situ polymerization of lactams. Pei et al. [98] prepared and studied PA-6/polystyrene (PS) (mass ratio range: 100/0–80/20), whereas Wu et al. [99] PA-12/PS blends (mass ratios: 100/0–75/25). In the former case styrene was polymerized in the CL melt, whereas in the latter work a premade PS was dissolved in LL melt. The polymerization temperatures were similar, 180 and 170 °C, respectively. PS formed the disperse phase until 15 wt % content. Above this threshold phase inversion (PA-6 and PA-12 became the disperse phase) took place. This is now the place to mention that the polymerization temperature was below the *T*_m_ of the corresponding PA but above the *T*_g_ of PS (~100 °C). Since the morphology is governed by the kinetics of phase separation, it is unstable and changes upon annealing, i.e., heat treatment above *T*_m_. This is a common feature of blends prepared by AROP of lactams. It is intuitive that the morphology is more stable when the interphase is “stabilized” by the compatibilizer.

Considering the fact that finely dispersed rubber domains with suitable interparticle distance are needed to initiate (via cavitation) and support the follow-up energy absorption mechanisms (occurring via crazing and/or shear yielding) [100], attempts were made to incorporate rubber modifiers during the in situ polymerization of lactams, as well. Omonov et al. [101] used liquid ethylene-butylene elastomer with terminal –OH groups as toughening agent. It was introduced into the CL melt with and without a previously prepared triblock copolymer PA-6-block-ethylene-butylene-block-PA-6). This triblock copolymer was synthesized by the above introduced macroactivator route. The PA-6/modifier content was set for 85/15 wt %. The Izod impact strength increased according to the range: PA-6 < PA-6 + hydroxyl functionalized ethylene-butylene elastomer < PA-6 + ethylene-butylene grafted PA-6 (macroactivator produced by reaction with isocyanate) < PA-6 + ethylene-butylene grafted PA-6 + triblock copolymer.

Yan et al. [102] prepared blends composed of PA-6, PS and maleated styrene-ethylene-butylene-styrene block copolymer rubber (SEBS-*g*-MA). The overall modifier content was constant (15 wt %), where the relative ration of PS to SEBS-*g*-MA varied. The Izod impact strength was improved along with a slight loss in stiffness and strength. This unusual behavior was traced to the peculiar formation of the dispersed domains in PA-6. The domains were composed of a hard core (PS) and a soft shell (ethylene-butylene and SEBS-*g*-MA segments in the interphase). These core-shell droplets were dispersed in submicron range.

PA-6 blends with styrene-acrylonitrile (SAN) and acrylonitrile-butadiene-styrene (ABS) copolymers have been developed and commercialized to reduce the moisture sensitivity, to improve the toughness and to reduce the shrinkage and warpage of PAs [103]. Therefore, trials were made to incorporate these polymers into PA-6 via in situ anionic polymerization of the latter.

Mohammadian-Gezaz and Khoshhal [104] prepared PA-6/ABS blends in the mass ratio ranges of 75/25, 50/50 and 25/75. Polymerization of CL, in which the ABS was dissolved, occurred at *T* = 200 °C. In order to improve the phase coupling, additional styrene-maleic anhydride (SMA) was introduced which was converted into a macroactivator. For comparison, purpose blends were produced by classical melt blending. The dispersed particles in the PA-6 matrix were smaller than those obtained by classical melt blending. The phase change (formation of bicontinuous morphology and phase inversion) was strongly affected by the relative amount of the micro- (isocyanate compound) and macro-activator (SMA-based growth center).

Hou and Yang [105] prepared PA-6/SAN (up to 15 wt %) blends by this in situ polymerization technique. The dispersion of SAN was much finer than in classical melt blending. The notched impact strength and tensile strength were ~50% higher than the reference cast PA-6 already at 2.5 wt % SAN content.

Wu et al. [106] produced PA-6/polymethyl methacrylate (PMMA, up to 10 wt %) blends which is a rather rare blend combination. Blends were proposed in two steps: radical polymerization of MMA in CL followed by the AROP of CL. PMMA was coarsely dispersed (≤5 μm) below 5 wt % PMMA content and phase inversion was observed already at 10 wt %. The PA particles received after dissolution of the PMMA phase exhibited a microporous structure.

Wu and Yang [107] incorporated a polymethacrylic ionomers in 0.5 and 1 wt % in PA-6 by in situ polymerization. These ionomers, capable to compete with the H-bonding between the PA chains, strongly decelerated the crystallization of the resulting PA-6. Parallel to that also the thermo-oxidative stability of the blends was reduced. Polyphenylene oxide (polyphenylene ether, PPE) is a traditional modifier of PS being miscible with it [103]. PPE was transformed into a macroactivator first by solution grafting with 4-methoxyphenyl acrylate followed by the reaction with NaCL in CL melt. Polymerization of the blends (CL/PPE ratio varied between 80/20 and 60/40) was done at *T* = 180 °C. The CL conversion was higher than 93%. Irrespective of the fact that CL was the major phase, the blends slowed an inverse morphology, i.e., PA-6 particles were dispersed in PPE [108].

Ahmadi et al. [109] polymerized CL in the presence of an acrylonitrile-butadiene rubber (NBR), adding up to 3 wt %. The Izod impact strength was tenfold while the stiffness and strength diminished only by ~20% at 3 wt % NBR content. According to transmission electron microscopic (TEM) pictures, the NBR particles were nanoscaled dispersed.

PA-6/TPU blends with up to 10 wt % TPU (polyether-type) were produced by Hou et al. [110] making use of the fact that the thermal dissociation of TPU in the CL melt yields –OH and –NCO functionalities and the latter may be involved in the macroactivator formation in the anionically polymerizable CL melt. Since in the etched cryofractured surface of the samples no voids could be revealed, the authors concluded the formation of a single phase structure, i.e., copolymerization occurred instead of blend formation. This conclusion was supported by dynamic-mechanical analysis (DMA).

Interchange reactions, such as transamidation between PAs [111], may be useful tools to improve their compatibility. This strategy was adopted by Wang et al. [112]. These authors incorporated a synthesized aliphatic–aromatic PA (*T*_g_ = 102 °C, *T*_m_ = 292 °C, up to 12 wt %) into PA-6 via in situ anionic polymerization of CL. The crystallized spherulites became finer by this modification. This resulted in improved toughness at the cost of stiffness and strength of these copolyamides. Note that the two latest referred works are in line with other introductory remarks that the terms blending and copolymerization may be “smeared”.

The processing techniques of the blends are similar to those listed to copolymers. It is noteworthy that research works have started also with the extrusion of different blends [113].

## 5. Nanocomposites

**Figure 6:**
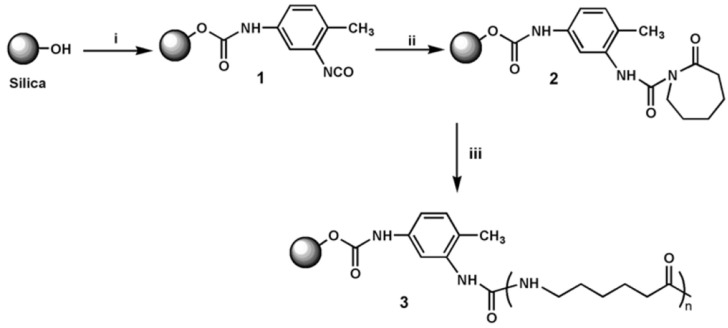
Synthesis of PA-6/silica nanocomposite in AROP via the “grafting from” approach. Designations: 1—toluene-2,4-diisocyanate (TDI), 2—CL-capping, 3—AROP of CL when initiated ([115] reproduced with permission from BME PT).

Different definitions exist for polymer nanocomposites which are also referred to as nanostructured and organic-inorganic (hybrid) composites. It is, however, generally accepted that the phase separated units, domains, and particles should be on nanoscale, at least in one space direction. Okada and Usuki [114] quoted that the term nanocomposite appeared from the 1990s. The “nanocomposite boom” started with the invention of polymer/clay nanocomposites, credited to Toyota Central Research Laboratories in 1985. Interestingly, the first matrix of the related nanocomposite was hydrolytically polymerized (also in situ) PA-6. The vast majority of polymer nanocomposites contain different fillers of inorganic origin. Their common feature is the high specific surface area. The nanofiller reinforcement can be produced ex situ (preformed particles) or in situ (sol-gel route, intercalation/exfoliation in the actual polymer). The nanofillers are usually grouped into (quasi)spherical, acicular or needle-like, and flaky or platelet-like types. These groups are frequently termed as 0 direction (0D), 1D, and 2D nanofillers, respectively. For their incorporation into polymers different methods may be adopted. From the viewpoint of technological applications in situ polymerization, melt compounding, and suspension-assisted techniques seem to be most suited. Since many nanofillers are of polar character and bear polar functional groups, such as –OH, –COOH, their surface should be rendered organophilic. Besides the traditional surface modification, AROP of lactams offers further possibilities. For example, the hydroxyl groups can be transformed into isocyanate which may form with the lactam an *N-*acetyl or *N*-carbamoyl group thereby generating an activator, growth center for the AROP. This approach is called “grafting from” since in this case grafting starts from the surface of the nanofiller—see Figure 6.

**Figure 7:**
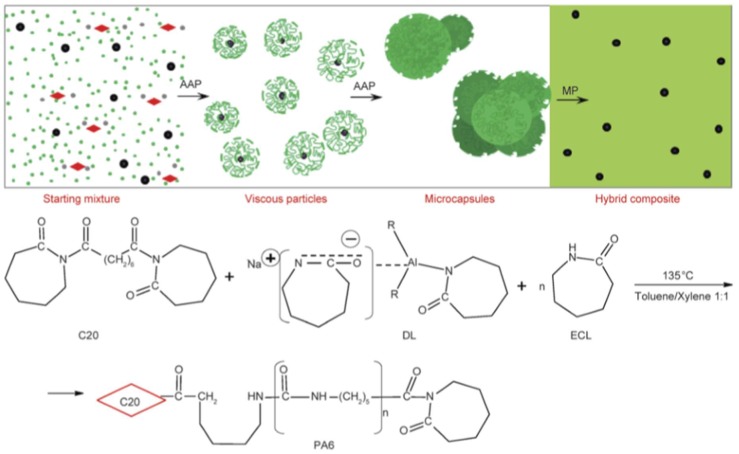
Morphology development of the nanocomposite during in situ solvent-assisted microcapsulation (top) and the related chemistry (bottom). Designations: APP—activated anionic polymerization, MP—melt processing above *T*_m_ of PA-6, C20—Bruggolen C20 activator, DL—Dilactamate^®^—dicaprolactamo-bis-(2-methoxyethoxo)-aluminate, ECL—ε-CL ([116] reproduced with permission from BME PT).

Next we summarize the achievements with polyamide nanocomposites produced in in situ AROP (Table 2). It is noteworthy that most of the related works synthesized the nanocomposites in casting and other melting techniques (rotational and extrusion molding). An important exception is given by the microcapsule technique shown in Figure 7.

When listing the relevant papers on PA nanocomposites we followed the grouping: 0D–1D–2D fillers. Due to the preferred applications of cast polyamides (tribological use) some microcomposites are also covered. It should be born in mind that the targets with the incorporation of nanofillers reinforcements are besides the improvements in structural properties (stiffness, strength, toughness) also the creation of functional ones (e.g., heat resistance, thermal [117] and electrical conductivity [118], magnetic behavior [119]). Reviews on PA-based nanocomposites [120,121] covered AROP produced versions only tangentially. Therefore, the present summary is likely the most exhaustive one in this field.

## 6. Composites

Due to a very low viscosity of cyclic lactams and superior mechanical properties of polymers obtained from them, these materials have a great potential for application in different liquid composite molding (LCM) techniques. It is not surprising that within the last 20 years vast academic research has been conducted to investigate possible industrial applications of anionically polymerized thermoplastic composites (TPC) reinforced with glass, carbon, aramid, or natural fibers. The first shots to produce such composites dated back to the 1970s and are related to the easiest LCM technique—casting. Further development resulted in more advanced technologies and some of them can be treated as new ones. Subsequently, nowadays a number of techniques are available for the production of anionically polymerized TPC parts. It is important to mention that huge progress has also been achieved in the development of machineries and materials [158]. This confirms a high level of overall industry readiness to adapt AROP for TPC production. The main advantages of such TPCs are their favorable mechanical properties, good ductility, recyclability, weldability, and compatibility with injection molding technique, that allows drastic parts’ integration and functionality enhancement [159,160,161].

Next we summarize the progress in different LCM techniques for the production of discontinuous fiber, continuous fiber (Table 3), and textile fabric (Table 4), reinforced anionically polymerized TPCs.

### 6.1. Discontinuous Fiber Reinforced

#### 6.1.1. Casting

**Figure 9:**
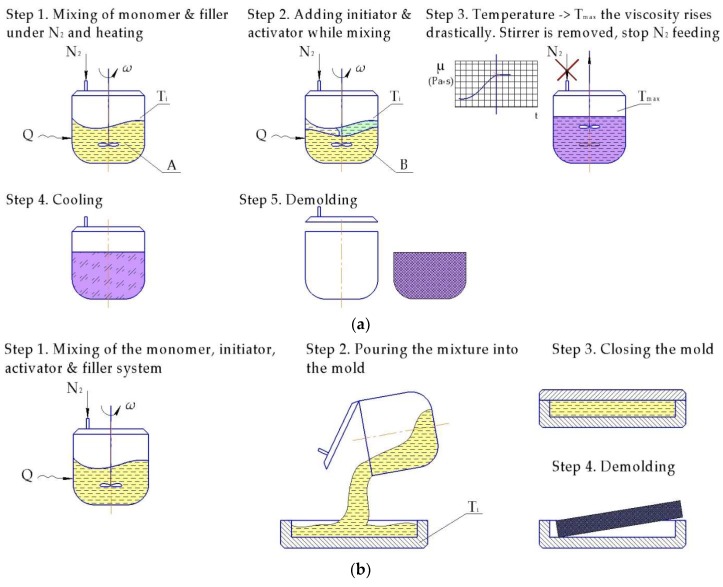
Schematic representation of the casting process for composite production: (**a**) in the same mold: A—Monomer & filler; B—Monomer & filler + initiator/activator; *T*_i_—initial temperature; *T*_max_—maximal temperature; (**b**) pouring into the mold from the vessel.

Casting is probably the earliest manufacturing technique used for the in situ polymerized composites (Figure 9). The main advantage of this manufacturing resides in simplicity and low capital costs. This process can be successfully used for the production of large parts.

One of the very first attempts to produce PA-6 reinforced with carbon fibers (CF) by casting was implemented by Litt and Brinkmann [162] in 1973. The authors used NaCL initiator and five different ester activators (tert-butyl acetate, ε-caprolactone, benzyl benzoate, benzyl acetate and phenyl acetate) for the AROP of CL filled with 20%–25% of short CF. They utilized a three-plate aluminum mold with a compression bar to form a composite at 220 °C within 30–40 min. Subsequently ε-caprolactone was selected as the optimal activator, since it delivered the best compromise between void content, reaction rate, and polymer quality. The more detailed description of the results is presented in Table 3.

The study dealing with the incorporation of short glass fibers (GF) into NBC matrix produced by casting was introduced by the Monsanto company in 1983 [163]. The purpose of the reinforcement application was improvement of mechanical and thermal properties of the resulting material. The authors used polyesteramide prepolymer with the acyllactam end groups acting as macroactivator for CL for the matrix and different types of reinforcement materials (1.65 mm chopped GF, mineral fiber, aluminum foil, and others). Among all the fillers the chopped GFs were considered to be the most efficient, as only 8% of them delivered 65% decrease in thermal expansion and more than 80% gain in the Young’s modulus (if compared with the reference unreinforced material). However, a 60% reduction in the impact strength was observed at the same time. At 25% loading of GF, a good balance of modulus and impact strength was achieved, while the thermal expansion level dropped to a suitable level. Another positive effect of GF incorporation was the increased resistance to expansion from moisture absorption.

Horsky et al. [164] investigated the effect of short GF, with and without (γ-aminopropyl)triethoxysilane sizing agent, on the PA-6 properties as well as the effects of the sizing concentration on the polymerization kinetics and mechanical properties of the matrix and corresponding composite. To polymerize CL, the authors utilized two initiators: (sodium dihydrido-bis(2-methoxyethoxo)aluminate and sodium tetra(6-caprolactamo)aluminate), while the cyclic trimer of phenyl isocyanate (PIC) was used as an activator. The reported results demonstrated that for a system with sodium dihydrido-bis(2-methoxyethoxo)aluminate/PIC the polymerization rate and the DOC, as well as yield strength and notched impact strength decreased, while the crystallinity and modulus remained unaffected. When the alternative system was used the polymerization rate was somewhat higher. The poor adhesion between fibers and matrix when using GF without sizing was also noted in the paper.

Engelmann et al. [132] found out that 15% of short CF in anionically polymerized PA-6 matrix (CL/C10/C20 system) improved tensile strength from 78 to 93 MPa (19%) and Young’s modulus could be doubled from 2660 to nearly 5399 MPa, at the same time reducing the degree of crystallinity. The authors also noted that the processability of the reinforced PA-6 was limited by the viscosity of the fiber containing monomer melt. As maximum a fiber content of 15% was quoted.

#### 6.1.2. Rotation Molding

**Figure 10:**
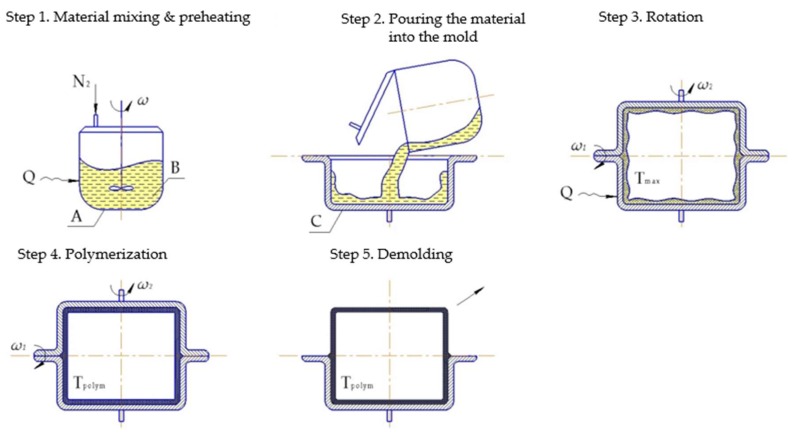
Scheme of centrifugal (without ω_2_) and rotation molding. A—dosing unit, B—monomer + initiator/activator + filler.

Rotational molding (Figure 10) is a polymer processing technique that has been widely used for the production of different tubes, wheels, pulleys, etc. The most commonly utilized materials for this process are dry powders. However, liquid resins have also been successfully used, thus enabling shorter cycle time and better properties of the resulting product. In that respect anionically polymerized lactams have high potential [45]. Incorporation of the fibers gives them the certain pros and cons of reinforced thermoplastics. However, proper processing parameters of the rotary molded reinforced PA-6 are an issue of great importance. Thus, Harkin-Jones and Crawford [90] investigated the influence of the initial mold temperature on the crystallinity degree and crystal size of rotationally molded Nyrim parts reinforced with GF. They observed that the crystallinity degree drops sharply if the initial mold temperature exceeds 140 °C, while the spherulite size increases. The authors mentioned that the incorporation of 5 mm length GF enabled improvement of the flexural properties, but with a decrease in impact strength.

### 6.2. Continuous Fiber Reinforced.

#### Pultrusion

**Figure 11:**
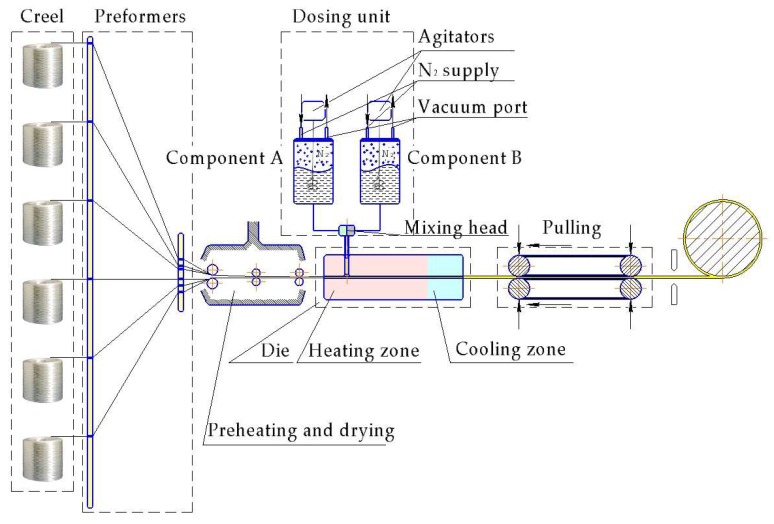
Scheme of pultrusion exploiting the in situ AROP of lactams.

Thermoplastic reaction injection pultrusion (TRI-pultrusion) (Figure 11) has become an issue of particular interest only at the present time. The interest is fueled by recent advances in material and equipment developments, as well as by the high commercial potential of this technique [165,166].

The main advantage of TRI-pultrusion is the utilization of fast reactive thermoplastic systems (CL, LL, CBT, etc.), which are all cyclic mono- or oligomers. Their use allows us to almost double the pultrusion speed. Several successful development projects in this field prove the feasibility of its industrial application. Nonetheless, no commercial TRI-pultrusion is available at present.

KraussMaffei (München, Germany) in cooperation with Thomas Technik (Bremervörde, Germany), Fraunhofer IGCV (Augsburg, Germany), Evonik (Essen, Germany) and Covestro (Leverkusen, Germany) developed a pultrusion system named iPul with the special injection unit that permits the use of fast reacting systems [165], yielding a production speed up to 3 m/min. Note that 0.5–1.5 m/min is the usual speed with thermosets. Another example is the CQFD Composites Company (Wittenheim-Alsace, France) that in cooperation with Hyundai (Seoul, Korea), Plasticomnium (Levallois Cedex France) and Arkema (Nanterre, France) developed pultruded front crash beam for Hyundai Motors (European Technical Center). This front crash beam contains unidirectional GF in anionically polymerized PA-6 matrix [167].

Apart from certain commercial benefits, the TRI-pultrusion allows unlimited length composite product with superior properties to be obtained, determined by the thermoplastic matrix and reinforcement. For example, transmission line conductor, traditionally consisting of steel, is subject to sag and failure because of the steel creep and hard operational conditions (such as high temperature gradients, icing, storms, etc.). Composite conductor made of anionically polymerized lactams or their copolymers and high modulus and strength long fibers can solve the described problems [168].

Nevertheless, the technological process development and process parameters optimization are essential issues and require comprehensive research activities. Luisier et al. [14,25,166] conducted a vast investigation on the development of the pilot pultrusion line for the in situ polymerization of LL. The development covered the fabrication of a mixing and injection unit, reinforcement pre-heating oven design, and evaluation of various die geometries, as well as determination of the processing window. Optimization of the process was aimed at maximizing the line speed, while achieving well impregnated TPC profiles. For detailed information, see Table 3.

### 6.3. Textile Reinforced

**Figure 12:**
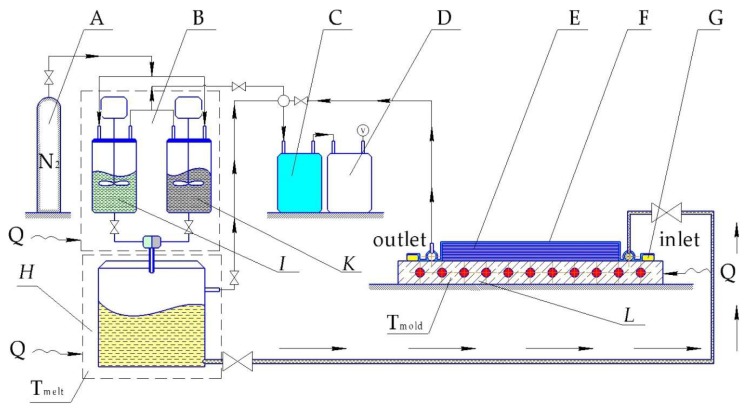
VARTM in situ polymerization process schematics: A—nitrogen source; B—dosing unit; C—cold trap; D—vacuum pump; E—textile preform; F—vacuum bag; G—sealing tape; H—buffer (degassing) vessel; I—tank with CL + initiator; K—tank with CL + activator; L—heated metal plate.

LCM techniques in case of in situ polymerization can include vacuum-assisted RTM (VARTM), thermoplastic RTM (T-RTM) or structural reaction injection molding (SRIM). In 2007 Rijswijk et al. [169] investigated whether the VARTM technique using GF-reinforced in situ polymerized CL is suitable to produce wind blades. The scheme of processing set-up is shown in Figure 12. Rijswijk stated that it is possible to produce huge parts with anionically polymerized PA6 matrices if the concentration of the activating system [170] and process parameters [171] are properly defined and controlled to reach the highest DOC, crystallinity, and mechanical properties [172,173]. It was proved that siloxyl groups on GF have an inhibiting effect and affect the interlaminar shear strength (ILSS) negatively. Therefore, proper aminosilane based sizing must be applied. It was also found that melt degassing has a crucial influence on the final void content. This issue has been investigated in depth already in the in situ polymerization of LL by Zingraff et al. [174]. Although the possibility of VARTM with AROP was proven for the wind blade production, it still has had no breakthrough and wide application. This is due to high risks imposed by the matrix system (sensitivity to moisture and UV irradiation), sophisticated tooling (achieving fast heating and cooling rates), and joining techniques for the blade halves as stated by Prabhakaran [175]. The work of Yan et al. [176] demonstrated the influence of different process parameters, like temperature, time, and activator concentration on the final product’s properties. Much less literature is available on CF textile reinforced CL-based composites produced via VARTM [177]. As long as the reduction of the production cycle time is the crucial parameter for the industry then two approaches are usually followed. In the first approach the polymerizable CL melt at 100 °C is injected into a mold with a temperature of 100 °C. Afterwards the mold is heated rapidly to 150 °C. In the second approach both the melt and the mold are already preheated to 150 °C. Although the second approach shows a possibility for cycle time reduction, it has a much shorter processing window which can lead to a not fully impregnated preform.

There are also some results with natural fiber reinforcements. Kan et al. [178] managed to produce ramie fiber-reinforced composite plates with sufficient DOC and crystallinity. The main challenge was related to the inhibition of the system due to the byproducts generated by the peeling reaction of cellulose in highly alkaline environment. Therefore, the less alkaline CLMgBr initiator was used and proved to be most suitable.

**Figure 13:**
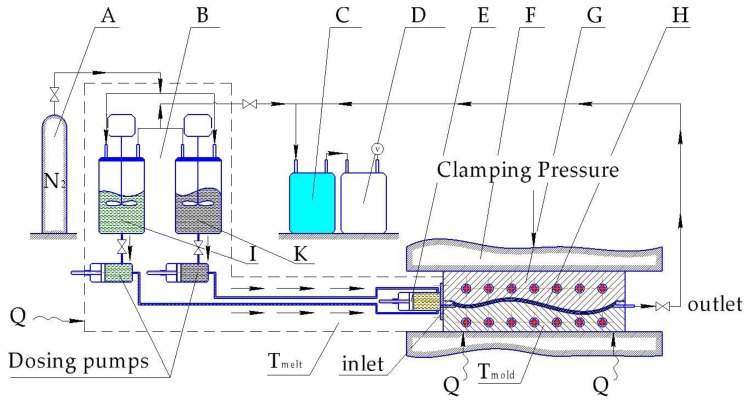
Scheme of the T-RTM process: A—nitrogen source; B—dosing unit; C—cold trap; D—vacuum pump; E—dynamic mixing head; F—mold carrier; G—metal mold; H—textile preform; I—tank with CL + initiator; K—tank with CL + activator.

Similar to VARTM, considerable investment is linked with the tooling system for the T-RTM process. The investment into equipment is a key issue for any composite parts manufacturer. The T-RTM process is outlined schematically in Figure 13.

Clamping units and dosing systems for T-RTM technology are high-end machines and can be very expensive. The more lines belonging to the dosing system the more expensive is the related unit (more sophisticated mixing control system and mixing head design). Thus Barfknecht et al. [179] demonstrated an innovative approach to implement a single-stream system with a soluble diisocyanate activator which was deposited onto the GF surface in advance. However, an extremely low DOC in the middle of the TPC plate was found as the result of the wash out effect generated by the monomer flow.

Successful industrial production of TPCs with textile-reinforced in situ polymerized CL was demonstrated by some companies, including KraussMaffei [158,180]. The hybrid (CF + GF) roadster roof cover frame was produced within a 2 min cycle time with a fiber volume fraction (*V*_f_) of almost 70% in 2016, and a B-pillar with 5-min cycle and *V*_f_ = 58% in 2014. The roadster project involved not only an injection but also a compression step, when excess amount of polymerizable CL was injected into the mold after the fiber impregnation was finished. It was foreseen to reduce shrinkage and void content, hence improving the surface quality. Such an approach was also followed in 2005 by Wakeman et al. [181]. A 0.2 mm gap was maintained by special spacers in the mold to provide enough space for injecting an extra 100 cm^3^ of melt, which was compressed afterwards with 55 bar overpressure. Another equipment producer, Engel (St. Valentin, Austria) successfully demonstrated its own developed CL dosing system with a unique in-mold mixing unit [182]. In their demonstration process also an overmolding step was implemented to enhance parts functionality [159]. For impregnation only 0.5–1.5 bar overpressure is mentioned as a requirement in the academic research, which makes possible using not only high pressure RTM dosing systems but also low pressure versions (LP-RTM). The latter are more cost efficient because of cheaper pumps and smaller clamping units. The compression molding phase is relevant for surface quality, especially when automotive parts are targeted (e.g., body-in-white concept for Pininfarina Nido car as described by Cischino et al. [183]). A CaproCAST process was introduced by Fundacion Tecnalia Research and Innovation (Spain) with GF reinforced PA-6 underbody element of the car. Cycle time of 6–8 min was achieved by an LP-RTM dosing system. Very poor paint adhesion was discovered and as a result it was concluded that PA-6/GF should be used for parts without aesthetical requirements.

Many research works have dealt with different testing methods to investigate mechanical properties, and especially the fracture and failure behaviors of TPC composites produced via AROP of lactams. In this respect non-destructive techniques such as acoustic emission and infrared thermography were used for detailed failure sequence analyses [184,185,186]. The summary of the state of the art for TPC reinforced with CF, GF natural fiber (NF) fabrics is shown in Table 4. Interestingly, the available sources state nothing about aramid fiber reinforced TPCs with in situ polymerized CL or LL.

### 6.4. Single-Polyamide Comosites

In single-polymer or self-reinforced composite (SPCs) both the reinforcing and matrix phases are given by the same polymer (one constituent), or by polymers belonging to the same family (two constituents). Albeit the concept “one polymer composite” was coined in 1975, it became a preferred research topic from 2000 onward [190]. SPCs are lightweight (the density of polymers is usually below that of traditional reinforcement) composites with ultimate recyclability (via remelting). ROP of monomers and oligomers are especially suited to produce SPCs through LCM operations. In respect of LCM the AROP of lactams features two major benefits: (i) the melt viscosity of the polymerizable lactam is very low, and (ii) the polymerization can be performed below the *T*_m_ of the resulting PA. Low viscosity is helpful to wet-out and impregnate the reinforcing structures. The difference between the polymerization and melting temperatures may be as high as 60 °C in the case of AROP of CL. This, so called “processing window”, is much larger than in other SPC production, where it is limited to a few degrees centigrade [190]. This large temperature difference is of great importance because the reinforcement otherwise loses its stiffness and strength with increasing temperature and dwelling time the closer the polymerization temperature is to the melting of the reinforcement. Last but not least, the fact that polymerization below *T*_m_ is accompanied by crystallization, allowing a faster demolding, is the guarantee for high productivity. In contrast to the above obvious benefits, PA-matrix based SPCs, prepared through activated AROP of lactams (exclusively CL) appeared only in 2010. Gong et al. [191,192] produced PA-6 (plane weave, in 65 wt % [191]) and PA-6,6 fabric (plain weave, in 60 wt % [192]) reinforced SPCs, respectively, via this LCM route. The in situ polymerized PA-6 impregnated well the reinforcement (void content <2.5%). Polymerization trials were made between 140 and 200 °C resulting in conversion >93%. The conversion was reduced with increasing temperature (linear/cyclic equilibrium) and in the presence of the PA-6 fabric reinforcement. This was attributed to the following effects: (i) impeded movement of CL toward the growth center within the reinforcing textile, and (ii) presence of H_2_O and –COOH groups on the PA-6 reinforcement surface which consumes –NCO groups of the activator used. Dencheva et al. manufactured PA-6 matrix based SPCs using PA-6 monofilament [193] and PA-6,6 textile [194] reinforcement, respectively. The content of the PA-6 fibers and PA-6,6 textile (plain weave), namely <20 wt % at <15 wt %, respectively, was quite low. As initiator sodium dicaprolactamato-bis-(2-methoxyethoxo)-aluminate, and as activator CL-blocked hexamethylene diisocyanate (Bruggolen C20P^®^), were used. To study the effect of sizing, they were removed by washing with acetone. The AROP of CL was performed between 160 and 170 °C [193] or at constant 165 °C [194]. The CL conversion measured was in the range 97%–99% and the *M*_v_ of the final PA-6 was ≥35 kDa. Based on detailed WAXS studies the authors confirmed the appearance of a transcrystalline layer (interphase between the matrix and reinforcement). Transcrystallinity is caused by laterally impeded spherulitic growth on closely spaced nuclei on a heterogeneous substrate (here the PA-6 or PA-6,6 reinforcements). Generally, the presence of a transcrystalline layer is considered to be a controlling factor of the stress transfer from the matrix to the reinforcement [195,196]. Interestingly, the thickness of this layer became smaller when the sizing was removed. The ultimate tensile strength and strain were enhanced with increasing reinforcement content, while only small changes were found for the stiffness. This was expected due to the low amount of the reinforcement and to the rather long dwelling time at the polymerization temperature. The *E*-modulus (stiffness) and strength of the reinforcing PA-6 and PA-6,6 diminished with increasing temperature and holding time. This is often accompanied by considerable shrinkage that can be limited by processing under pressure when applicable (see below).

In the recent paper Dencheva et al. [197] produced PA-6-based SPCs with dual reinforcements. The reinforcement contained organophilic montmorillonite clays (OMMT) and PA-6 fabric layers. The PA-6 matrix, with and without MMT (1 wt %), was prepared by the former introduced micro-capsulation (MC) technique. PA-6 MCs were synthesized in solution adapting the activated AROP of CL, before precipitating and drying to receive micronscaled MCs. The PA-6,6 fabric layers were powder-impregnated by these MCs prior to compression molding at *T* = 230 °C under 5 MPa pressure for 10 min. Note that the compression molding temperature was below the *T*_m_ of the PA-6,6 reinforcement. The volume fraction of PA-6,6 fabric in the SPCs was between 0.3 and 0.6. The beauty of this technique is that the MMT particles are well and uniformly dispersed. By contrast, direct impregnation of the reinforcement with a polymerizable CL melt containing MMT may result in a filtering-off of the particles owing to the dense mesh structure of the fabric layers. In this way an MMT-rich surface layer may appear which is undesirable. Tensile tests showed that MMT alone embrittled the PA-6. The combined use of MMT + PA-6,6 (dual reinforcement) worked for enhanced ductility with strain-hardening phenomenon. The stiffness and ultimate tensile strength of the SPCs with dual reinforcement were 66% and 73% higher than the reference PA-6 at 0.3 volume fraction reinforcement. On the other hand, a prominent (~fivefold) increase was found with respect to the toughness. At higher PA-6,6 fabric content the tensile mechanical properties dropped owing to poor impregnation. Unexpectedly, this had only a marginal effect on the toughness.

## 7. Outlook and Future Trends

Based on this review the following conclusions can be deduced:

- CL remains the preferred monomer for AROP and in the future. The present commercially available initiators and activators will be used in the near future, and attempts will be made to reduce their “environmental” (i.e., humidity) sensitivity. Solvent-borne, liquid initiators and activators may likely be preferred. Search for effective latent initiators/activators will be under the spotlight of research in academia targeting the use of a one-component system that is prone to “polymerization on demand”. The copolymerization strategy will further focus on the toughness improvement of the related PA-6-based (block) copolymers. Besides the traditional block segments (polyether- and polyester-based diols) others, like polycaprolactone, polylactic acid etc. may be incorporated in order to enhance the renewable content and support biodegradability. The in situ blending via AROP will hardly achieve industrial breakthrough.
- Vigorous development can be predicted for PA-6-based nanocomposites produced through AROP thereby making use of the “grafting from” (i.e., transforming the surface of the nanofillers into a suitable “nanoactivator” for grafting the CL chains) approach. This development will target the production of new tribological compounds, containing novel carbonaceous nanofillers, which will be most likely produced still by casting. The toughness of such nanocomposites will be a key factor and thus the related works will be supported by extensive modeling [198]. According to our view, novel and adapted manufacturing methods will be the real future drivers of the development of thermoplastic composites with AROP-produced matrix. Additive manufacturing via ink jetting should be mentioned among the emerging novel techniques. Among the “adapted” techniques a bright future can be predicted for thermoplastic reaction injection pultrusion (TRI-pultrusion), thermoplastic resin transfer molding (T-RTM), and other liquid composite molding procedures. This claim is based not only on the straightforward recyclability of the related composite parts, but also on other beneficial design- and post-processing-related features, such as part integration, overmolding (with and without additional reinforcements), surface coating and finishing, and welding. The related research and developments works will run parallel with extensive modeling (especially via finite element codes) studies. The potential of PA-6-based single-polymer (self-reinforced) composites has been strongly underestimated, therefore in this field interesting developments may be expected.

## Figures and Tables

**Figure 8:**
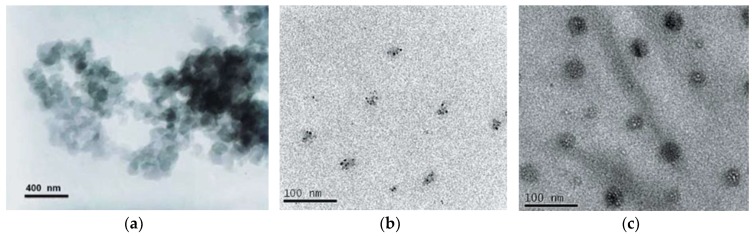
Representative TEM images of pristine silica (**a**), and PA/silica nanocomposites with different silica mass fractions: 2 wt % (**b**), and 10 wt % (**c**), respectively ([115] reproduced with permission from BME PT).

**Table 1:** Processing and material parameters of specimens produced by classical and reactive rotational molding [46].

Rotational Molding	Classical	Reactive
Temperature	*T* ~ 240 °C	*T* ~ 150 °C
Cycle time	*t* >40 min	*t* = 15–20 min
Speed ration (*S*_1_/*S*_2_)	5/4	5/4
Melting point *T*_m_ (°C)	224.3	224
Degree of crystallinity (%)	28	49
Degree of conversion (%)	98.9%	98.9%
Intrinsic viscosity (dL/g)	1.07	7
Molecular weight (g/mol)	30,778	182,594
**Tensile Properties**		
Young’s modulus (MPa)	750	1560
Yield stress (MPa)	62	80
Elongation at break (%)	32	64

**Table 2:** Preparation and properties of PA nanocomposites through in situ AROP. Notes: when different fillers were used all of them are mentioned at the related reference. Relevant works using micron-scale fillers are also included.

Nanofiller (Type, Amount)	Monomer	Initiator/Activator (Type, Amount)	Preparation	Testing	Results, Comments	Refs.
Cu-, Zn-, Fe- particles (micron-scale, up to 8 wt %) Al- particles (micron-scale, up to 30 wt %)	CL	Dilactamate^®^/C20 (3 mol %/1.5 mol %)	In solvent (toluene/xylene = 1:1) at 135 °C, followed by filtering, drying and compression molding at *T* = 230 °C	Optical microscopy, viscosimetry (*M*_v_), DSC, TGA, synchrotron WAXS, electric conductivity, tensile tests	PA-6 microcapsules (see Figure 7) produced and their formation mechanism proposed. PA-6 nanocomposites exhibited enhanced *E*-modulus and tensile strength. γ-polymorph formed upon nanofiller loading. No electric percolation observed. Potential for energy storage deduced.	[121]
SiO_2_ (7 nm) with and without silane surface modification (2–10 wt %)	CL	Dilactamate^®^/*N*,*N*′-[methylene-di(4,4′-phenylene)bis-carbamoyl]bis-ε-caprolactam (0.8 mol %/0.4 mol %)	CL with initiator +CL with activator mixed separately at *T* = 100 °C. Two melts mixed and polymerized at 160 °C for 40 min via rotational molding	Viscosimetry (*M*_v_), water uptake, DSC, TGA, WAXS, TEM, FTIR, impact and flexural properties	Silane treatment of silica improved the polymer yield (>95%), reduced the water absorption, enhanced the flexural modulus and strength. Notched Izod impact strength (IS) (peaked at 4 wt %) was also improved by contrast to the unmodified silica.	[122]
Porous SiO_2_ (20 nm) functionalized with TDI (<14 wt %)	CL	Na/SiO_2_ with carbamoyl and group (see Figure 6) (molar ratio = 6:1)	CL + initiator + activator mixed at 80 °C under N_2_ and sonicated for 30 min. Polymerization at 170 °C for 6 h will varies feed ratios	Viscosimetry (*M*_v_), FTIR, DSC, TGA, TEM	Feed ration CL/(initiator + activator) affected *M*_v_ reaching ~12 kDa. TGA proved the “grafting from” approach, i.e., CL polymerization according to the scheme in Figure 6. At higher SiO_2_ content prominent agglomeration found (Figure 8)	[115]
SiO_2_ (5 μm) acicular—aspect ratio ~15 with amino coupling agent	CL	NaOH/isocyanate (TDI)	Particles introduced in CL melt at 130 °C. NaOH added upon stirring for 30 min followed by dosing TDI. Cast polymerization at 170 °C for 20 min	FTIR, DMA, DSC, WAXS, SEM, mechanical properties	Particles well dispersed. Tensile and notched Charpy IS increased, reaching a maximum at 3–5 wt % silica, then decreased. Nucleation and crystallization affected by the silica presence. Silica needles pulled out thereby enhancing the toughness	[123]
TiO_2_ (<10 μm) with and without surface treatment with aminosilane ≤ 8 wt %	CL	Dilactamate^®^/*N,N′*-[methylene-di(4,4′-phenylene)bis-carbamoyl]bis-ε-caprolactam (0.8 mol %/0.4 mol %)	Polymerization via rotational molding at *T* = 160 °C for 30 min	DSC, TGA, tensile properties, notched Izod	Tensile and flexural moduli increased with increasing filler content without any effect of surface treatment. The latter surface treatment improved the strength. The toughness and tensile elongation were reduced with increasing TiO_2_ content whereby marginal effect of silane coupling was observed	[124]
Metals, Metal oxides, Carbon black (CB), Graphite, CNT, CNF Organoclays	CL	Dilactamate^®^/C20 (3 mol %/1.5 mol %)	Microcapsules’ production in solvent (see Figure 7). Capsules filtered, dried and specimens produced by compression molding at *T* = 230 °C at 5 MPa pressure	Optical microscopy, SEM, mechanical properties, electric and magnetic behavior	Conversion up to 85% and filler content (“pay-load”) up to 30 wt %. Mechanical and dielectrical properties tailored upon amount, type and combination of the additives.	[125]
Yttrium hydroxide with and without surface treatment, diameter: ~400 nm, length: few microns (<0.8 wt %)	CL	NaOH/TDI	Cast polymerization at *T* = 180 °C for 1 h	SEM, WAXS, tensile and impact testing	Good dispersion of the filler. Tensile strength and water absorption reduced, whereas impact strength increased, peaking at ~0.3 wt %	[126]
Boron carbid (B_4_C) 15–62 μm, Graphite ~10 wt %	CL + isophorone diisocyanate functionalized polypropylene-glycol (PPG) macroactivator (NBC-type)	NaCL/macroactivator	Bulk polymerization in ampule and in mold casting. Mixing with filler, in situ macroactivator preparation at 120 °C under N_2_. Initiator added at 140 °C. Polymerization at 180 °C.	Degree of conversion (DOC), ^1^H-NMR, FTIR, Charpy impact	Copolymer formation between CL and PPG verified. At high macroactivator content polymerization rate and yield are influenced by the filler (B_4_C and graphite). Charpy IS strongly improved but its change with the filler content differed between B_4_C and graphite	[127]
POSS with –NH_2_ functionality (≤16 wt %)	CL	NaH (NaCL)/cyclohexyl-carbamoylcaprolactam or POSS-CL (reaction product of POSS-NH_2_ with carbonylbiscaprolactam, activator content varied between 0.6 and 1.8 mol %)	Different polymerization techniques: hydrolytic, quasi-adiabatic AROP, isothermal AROP, anionic suspension polymerization	DOC, viscosimetry (*M*_v_), DSC, WAX, SEM	In AROP techniques DOC was higher than 93%, *M*_v_ varied between 13 and 176 kDa as a function of AROP technique and activator type/amount. AROP performed better than the hydrolytic route. The tensile behavior of the POSS-containing nanocomposites featured improved ductility at cost of stiffness and strength	[128,129]
CB, MWCNT, CNF, Graphite (≤10 wt %)	CL	Dilactamate^®^/C20 (3 mol %/1.5 mol %)	Microcapsules production in solvent—see Figure 7. Capsules filtered and dried prior to compression molding	Optical microscopy, viscosimetry (*M*_v_) DSC, TGA, synchrotron WAXS electrical, dielectrical behavior	All fillers enhanced the stiffness and reduced the deformation at break with increasing content. Tensile strength improvement was found out for MWCNT. The conductivity, permeability strongly changed as fraction of the type, amount and combination of these fillers	[116]
C_60_ (fullerene) [6,6]phenyl-C_61_-butyric acid methyl ester (≤3 wt %)	CL	Dilactamate^®^/C20	Modified fullerene dispersed in molten CL at 110 °C under N_2_ blanket. Then initiator/activator introduced, homogenized and polymerized at *T* = 170 °C for 30 min	DOC, viscosimetry (*M*_v_) DSC, TGA, DMA, FTIR, SEM, WAXS, electric conductivity	Complex interaction between the π-electrons of fullerene and CL revealed that strongly effected the polymerization. Formation mechanism for the linear/crosslinked chain formation proposed. The volume resistivity above 0.1 wt % fullerene content was reduced by 2–4 order of magnitude	[130]
C_60_, C_60_/C_70_ mixture, fullerene soot (0.5–2 μm)	CL	Na/toluene-2,6-diisocyanate	Bulk polymerization at *T* = 140–160 °C for 12 h	DSC, electrical resistivity, tensile and compression properties, tribology	Small enhancement in stiffness and strength with increasing fullerene content. Volume resistivity decreased with 6 order of magnitudes at a fullerene content of 0.10 wt %. The coefficient of friction was halved in presence of fullerenes.	[131]
CB nanoscale SiO_2_ micronscale SiC submicronscale SCF ≤ 15 wt %	CL	C10/C20 also in presence of a curing agent for electron beam irradiation	Filler introduced in the activator-containing CL at 120 °C. AROP performed at 160 °C for 30 min	Viscosimetry (*M*_v_) DSC, TGA, heat distortion temperature (HDT), DOC, SEM, TEM, mechanical testing	Stiffness, strength and HDT improved in the range of 10%–30% at 2 wt % filler content. 15 wt % short carbon fibers (SCF) enhanced the tensile strength from 78 to 93 MPa and doubled the *E*-modulus. Crystallinity slightly reduced. Effect of the dose of electron beam irradiation was moderate for nanoscaled CB.	[132]
Graphite (colloidal) with and without titanate coupling agent 4 μm ≤ 8 wt %	CL	NaOH/TDI (0.5 mol %/0.5 mol %)	Filler dispersed in molten CL at 130 °C before adding the initiator and activator under vacuum. Cast polymerization at 175 °C for 30 min	Viscosimetry (*M*_v_), FTIR, DSC, WAXS, DMA, mechanical properties, friction/wear	MW reduced from 85 to 55 kDa with the graphite content. Graphite worked as heterogeneous nucleant during crystallization. The tensile strength did not change until 4 wt % Graphite before drastic reduction. Notched Charpy IS improved only at 0.5–1 wt %. PA-6 with 4 wt % graphite exhibited more than 10-fold increase in wear resistance.	[133]
Graphite, 5 μm (5 wt %)	CL	Dilactamate^®^/PUs (2/1; 1.8/0.8)	CL molten under N_2_ and mixed with the PU (macroactivator), followed by introduction of the graphite powder and the initiator. Casting at 170 °C for 1 h	Optical microscopy, DSC, DMA, tensile tests, flexural creep, tribology	Composites with gradient structure produced. Polyether-urethane as macroactivator yielded high MW with crosslinking. Graphite filling reduced MW, the spherulite diameter, the tensile strength, elongation at break and the coefficient of friction (by 50%)	[134]
SWCNT functionalized with CL	CL	Na/CL-functionalized SWNT	Polymerization at *T* = 140 °C for 24 h.	SEM, ^1^H-NMR, Raman spectroscopy, TGA, AFM, UV spectroscopy	“Grafting from” approach. i.e., covalent bonding of CL to CNT followed by the AROP of CL, proved	[135]
MWCNT (<0.3 wt %)	CL	Dilactamate^®^/TDI (0.3 mol %/0.15 mol %)	CL mixed with MWCNT at 100 °C, then initiator introduced at 135 °C followed by the activator and mixing. Cast polymerization at 175 °C for 3.5–4.5 min.	DOC, DSC, TGA, DMA, mechanical properties	DOC >96%. All nanocomposites showed increased tensile modulus and strength compared to neat PA-6. The elongation at break did not change whereas the Charpy IS decreased with increasing MWCNT content	[136]
MWCNT with –OH functionality	CL	Na(NaCL)/CL-functionalized MWCNT (MWCNT-OH reacted first with TDI and then with CL), see Figure 6	CL + Na + CL-functionalized MWCNT mixed/sonicated at 70 °C for 30 min. Polymerization at 170 °C for 6 h	FTIR, TGA, UV-Vis, TEM	“Grafting from” approach in two steps (CL-functional MWCNT activator formation and acyl-CL initiated AROP of CL) confirmed	[137]
MWCNT (purified) <1 wt %	CL	NaH (NaCL)/*N*-acetyl-caprolactam	CL + polyoxyethylene + MWCNT + acetyl-caprolactam mixed/sonicated, then NaH added and polymerized at 120 °C for 6 min. Fibers produced at different stretching ratios.	Viscosimetry (*M*_v_), SEM, DSC, tensile tests	MWCNT dispersed by ultrasonication. Tensile *E*-modulus and tensile strength increased by ~40% in case of 1 wt % MWCNT containing nanocomposite with a stretching ratio of 4.	[138]
MWCNT with –OH functionality (≤0.2 wt %)	CL	NaCL/TDI	MWCNT-OH dispersed in molten CL through a water-assisted method. Water removed at 170 °C. Then NaCL and TDI introduced, polymerization at 160 °C for 10 min.	Optical microscopy, DSC, TEM, TGA	Fine dispersion of MWCNT-OH acting as heterogeneous nucleating agent. DOC ~96%	[139]
MWCNT with –OH functionality (≤1.5 wt %)	CL	NaCL(C10)/MWCNT-NCO + TDI (prepared by reacting MWCNT-OH with TDI)	To CL solution in DMF MWCNT-NCO was added and ultrasonicated at RT. DMF removed in vacuo and heated to 170 °C. After adding TDI, NaCL was added and cast polymerization performed at 160 °C for 10 min.	FTIR, SEM, DSC, TGA, tensile properties	PA-6 chains covalently attached to the sidewalls of MWCNT which were uniformly dispersed. MWCNT worked as nucleating agent and also improved the thermal stability. Tensile modulus and strength were markedly improved at cost of the elongation at break.	[140]
MWCNT	CL	C10/C20 (0.3 wt %/0.3 wt %)	Small samples produced for DSC and rheology tests at *T* = 180–220 °C	DOC, DSC, design of experiments, GPC, rheology	MWCNT had inhibiting effect on the AROP of CL. DOC was simulated. The MW was not affected by MWCNT. It was suggested that MWCNT may react with the initiator.	[141,142]
MWCNT (≤5 wt %)	LL	NaH/*N,N′*-ethylene bis(stearamide) (molar ratio = 1/0.5)	Polymerization in microcompounder: premixing at 170 °C for 5 min and polymerization at 270 °C for 4 min under N_2_	TGA, GPC, optical microscopy, TEM, electrical conductivity	DOC at ~99%. *M*_v_ values between 10 and 41 kDa along with polydispersity in the range of 1.5–2.2. MWCNT delayed the polymerization. Volume resistivity dropped 8 order of magnitudes at 5 wt % MWCNT compared to the neat PA-6. Similar results obtained by classical melt mixing of PA-12 with MWCNT.	[143]
MWCNT (1 wt %)	CL + styrene (successive polymerization, styrene first) PA-6/PS blend ratio: 80/20	NaCL/TDI	First PS/CL/MWCNT mixture obtained after the polymerization of styrene. To this mixture NaCL and TDI were added at 150 °C and residual styrene removed. Cast polymerization of CL at 180 °C for 20 min.	SEM, TEM, dielectric spectroscopy	PS became the dispersed phase and MWCNTs were selectively located in the interphase between PA-6 matrix and PS.	[144]
CNF (stacked-cup) ≤ 0.8 wt %	CL	Na (NaCL)/caprolactam-functionalized CNF + caprolactam-capped diisocyanate	CNF was acid treated and functionalized with HMDI in DMF, then capped with CL. CL melted at 80 °C and CL-functionalized CNF + CL-capped diisocyanate added. Polymerization at 150 °C for 30 min.	Viscosimetry (*M*_v_), TEM, FTIR, TGA, SEM, PSC, WAXS, mechanical and impact tests	Stiffness and strength significantly enhanced along with slight improvement in toughness. CNF promoted the formation of the γ-phase. *M*_v_ data scattered between 54 and 59 kDa.	[145]
Cellulose nanocrystal (CNC) (≤2 wt %)	CL	NaH (NaCL)/phenyl isocyanate (1.5 mol %/1.2 mol %)	CNC dispersed in molten CL under sonication. Initiator added in N_2_ atmosphere. Activator, prepared separately by reacting CL with the isocyanate, added and polymerization at 150 °C for 30 min.	DOC, TGA, DMA, AFM, SEM, creep melt rheology	CNC was efficient reinforcement: improved the creep resistance, enhanced the DMA properties. The zero shear viscosity was prominently higher in CNC presence compared to the neat PA-6, suggesting the onset of a percolated structure that was prone for breaking upon shear.	[146]
CNC with and without aminosilane surface modification (≤3 wt %)	CL	EtMgBr (CLMgBr)/C20	CL + CNC +initiator was mixed with CL + activator and polymerized at 150 °C. Samples produced by extrusion. For comparison purpose classical melt blending served.	SEM, TEM, TGA, FTIR, solid state NMR, rheology (nano) mechanical tests	Based on solid state NMR CNC-grafted PA-6 was proposed (involving transamidation, urea bond formation). Tensile stiffness and strength strongly improved at cost of elongation at break. Melt elasticity and strength enhanced by CNC reinforcement.	[147]
MMT, pristine (NaMMT) and organophil (intercalant: dioctadecyl dimethyl ammonium chloride) versions (OMMT) (≤2 wt %)	CL	C10/TDI	NaMMT dispersed in aqueous CL under ultrasonication. Afterward water removed in vacuo at 170 °C, then initiator added followed by TDI. Polymerization at 160 °C for 10 min. OMMT introduced directly or in acetone—assisted dispersion.	GPC, X-ray diffraction (XRD), TEM, TGA, DSC	DOC was higher than 94% except OMMT (86%). *M*_n_ and *M*_w_ values were at about 20 and 50 kDa respectively. NaMMT was exfoliated based or XRD results below 1.5 wt % content. Above this intercalation took place. The thermal stability was prominently improved by NaMMT. NaMMT acted as heterogeneous nucleant and promoted also the γ-phase formation. OMMT appeared in intercalated structure and did not improve the PA-6 matrix properties.	[148,149]
NaMMT (pristine clay) (3 wt %)	CL	NaCL/TDI in presence PMMA-Na^+^ ionomer as compatibilizer	NaMMT + CL + PMMA-Na^+^ ionomer mixed in aqueous solution, then water evaporated. Initiator and activator added and cast polymerized at 180 °C for 10 min.	XRD, DSC, TEM, shear viscosity	NaMMT was intercalated in absence of the compatibilizer or in its low amount. Exfoliated structure received in the blend PA-6/clay/ionomer = 97/3/4.5. Well dispersed clay layers reduced the crystallinity and favored the formation of the γ-polymorph.	[150]
Clay (MMT) with and without organophile modification (≤4 wt %)	CL	Initiator/activator undefined	Preparation via reactive extrusion. (CL + initiator) and (CL + activator) were separately introduced into an extruder. Extruder temperatures: polymerization and processing zones 180 °C and 220 °C, respectively. Clay added differently.	TEM, optical microscopy, tensile properties	Continuous production of PA-6/clay nanocomposites is feasible. Clay particles are intercalated/partly exfoliated. The *E*-modulus of PA-6 is increased by 20% and 30% by the incorporation of 2 and 4 wt % clay, respectively.	[151,152]
NaMMT (clay) (2 wt %)	CL, LL, CL + LL	NaCL or CLMgBr/*N-*acetyl caprolactam (0.5 mol %/0.5 mol %)	AROP of lactams performed at 180 °C for 30 min in N_2_ atmosphere	DOC, GPC, XRD, DSC, SEM, TEM	NaCL produced random, whereas CLMgBr tended to result in block copolymers. The intercalation was reduced with increasing LL content. In the block-type copolymer the intercalation of clay remained the same with increasing LL content. LL content reduced the DOC and MW of the final copolymer. Crystallinity strongly reduced by LL content.	[153]
OMMT (≤10 wt %)	CL	Dilactamate^®^/C20 (1.5 mol %/0.75 mol %)	CL melt mixed with OMMT under N_2_ at 110 °C. Then initiator and activator added. Polymerization in a mold placed in a hot press (165 °C, 10 MPa)	DOC, Synchrotron WAXS, FTIR, TEM	Conversion > 97%. Up to 1 wt % OMMT was exfoliated, above this intercalated. Micronscale OMMT agglomerates also revealed. The matrix in the nanocomposites was α-phase. After melting/recrystallization the γ-form appeared.	[154]
OMMT (≤10 wt %)	CL	NaH/*N-*acetyl caprolactam	Polymerization in solution using NMP at 160 °C for 30–45 min	DSC, SEM, WAXS, viscosimetry (*M*_v_)	MW dropped with increasing OMMT content. Crystallinity increased up to 1 wt %. OMMT then decreased. At higher OMMT content PA-6 crystallized in γ-form. OMMT intercalation was supported by the polymerization in solvent.	[155]
Graphene (≤0.5 wt %)	CL	NaOH/TDI	Graphene added to molten CL and ultrasonicated. NaOH introduced and water removed in vacuum at 180 °C followed by dosing TDI. Cast polymerization at 160 °C for 15 min	GPC, TEM, SEM, XPS, Raman, DSC, TGA, mechanical properties	MW (both *M*_n_ and *M*_w_) slightly reduced with increasing graphene content. Nanocomponents displayed higher thermooxidative stability than PA-6. Flexural modulus, strength and impact strength drastically enhanced while the formation of γ-polymorph promoted.	[156]
Graphene oxide (GO) (≤1 wt %)	CL + ε-caprolactone (ratio: 90/10 and 80/20)	CLMgBr/ε-caprolactone (activator)	GO dispersed in molted CL at 80 °C in Ar atmosphere. Mixture heated to 110 °C and initiator added, followed by ε-caprolactone. Cast polymerization at 150 °C for 1 h.	XPS, TGA, TEM, viscosimetry (*M*_v_) DSC, mechanical tests	*M*_v_ decreased with GO content. The formed poly(ester amid) was random type. GO acted as nucleating and reinforcing additive. *E*-modulus increased while impact strength decreased with increasing GO content.	[157]

**Table 3:** Preparation and properties of short/continuous fiber reinforced composites via in situ AROP.

Reinforcement	Monomer/Solvent or Copolymer (Amount)	Initiator/Activator (Amount)	Technology	Process Parameters	Testing	Results, Comments	Refs.
GF	CL/-	Sodium dihydridobis(2-methoxyethoxo)aluminate/PIC (0.3/0.3 mol %)	Casting	*T*_polym_ = 135 °C; *T*_0_ = 133–134 °C; *T*_max_ = 205 °C;	*ρ*, X_C_ (X-ray diffraction), *V*_f_, SEM, DMA, mechanical tests, DSC	- Properties of PA-6 are affected by rising concentration of sizing agent: polymerization rate, σ and IS decrease, DOC rises; X_C_ and Young’s modulus remain unaffected. - GF increase modulus, but do not affect the time dependence of the creep in the interval 10^−1^–10^4^ min. - Rising fraction of GF: decreases σ (which indicates poor adhesion between matrix and GF), decreases IS, while the opposite trend can be seen for silane treated GF.	[164]
		Sodium tetra(6-caprolactamo) aluminate/PIC (0.3/0.3 mol %)					
	CL/-	NaCL/HMDI (0.75/0.75 mol %)	Pultrusion	*V*_f_ = 72 %; *T*_proc_ = 140–160 °C; *V*_pul_ = 40.6 cm/min; *T*_react_ = 52 s.	FTIR, DMS, viscosity, IS, SEM	- The Nylon-6 reaction is finished in 52 s at 160 °C. - The possibility of engineering the composite impact failure behaviors by using rubber-toughened matrices to achieve a higher toughness is illustrated.	[91]
	LL/Dimethylpropylene urea	NaCL (0.75 wt %; 1 wt %; 3 wt %)/ cycloaliphatic monocarbodiimide (0.75/0.75 wt %) (1/1 wt %) (3/3 wt %)	Pultrusion	*T*_proc_ = 230–290 °C *V*_pul_ = 0.8–3.4 m/min *d*_polym_ = 3.15 m; 2.10 m; 1.05 m. *F*_pul_ *= f (T*_proc_*) =* 300–1450 N	DOC, X_C_	- Optimization of the thermoplastic pultrusion process was performed (pulling speed → max, while achieving impregnated and polymerized profiles). - Maximum die lengths were determined by the evaluation of pulling forces. - A processing window has been defined in terms of pultrusion line speed and mold temperature.	[25,166]
	NBC	Not mentioned/acyllactam end groups & carbonyl groups of the polyesteramide prepolymer	Casting	*T*_polym_ = 130 °C	Mechanical properties, thermal expansion, water absorption.	GF in NBC gives increased resistance to expansion from moisture absorption and thermal changes. Temperature resistance of stiffness and resistance to heat sag improved. Losses in IS may be partially restored by moisture absorption and/or changes in resin matrix formulation.	[163]
	NBC	Acyllactam end groups of the prepolymer */not mentioned *Polyesteramide prepolymer terminated by acyllactam	Rotation molding	*T*_mold_ = 110–190 °C	X_C_ = *f* (*T*_mold_); IS = *f* (*T*_mold_, *t*_cycle_, filler); *E* = *f* (*T*_mold_, filler); Shrinkage = *f* (*T*_mold_); Water uptake = *f* (*T*_mold_)	- Initial *T*_mold_ has a great effect on the X_C_ and crystal size. X_C_ drops sharply as initial *T*_mold_ > 140 °C, while spherulite size increases. - The IS increases with the *T*_mold_. - Slight increase in the X_C_ with the increase of the oven cycle time. - The IS of Nyrim parts decreased as filler was added while the flexural properties are improved.	[90]
CF	CL/-	NaCL/tert-butyl acetate (1/2 mol %)	Casting	*t*_react_ = 30–40 min; *T*_polym_ = 195–220 °C	Mechanical properties	- Caprolactone was selected as activator (best compromise between void content, reaction rate and polymer quality). - Benzyl acetate and benzyl benzonate produced very slow reaction, though without voids. - Tert-butyl acetate caused rapid reaction and a very tough polymer, but with many voids. - Phenyl acetate worked for fast reaction yielding good polymer product, but it can terminate the reaction if used in excess. - Casting is difficult with *V*_f_ > 35%.	[162]
		NaCL/ε-caprolactone (1/2 mol %)					
		NaCL/benzyl benzoate (1/2 mol %)					
		NaCL/benzyl acetate (1/2 mol %)					
		NaCL/phenyl acetate (2/1 mol %)					
	CBT, AROP of lactams and their copolymers	Not disclosed	Pultrusion	3 heating zones in the die: *T*_1_ = 170 °C, *T*_2_ = 180 °C, *T*_3_ = 190 °C. *T*_oven_ = 240 °C	*E;* σ; ε_R_	The field of the invention relates to the conductor for electrical transmission lines having composite load bearing core produced by pultrusion using a thermoplastic polymer matrix, by in situ polymerization of the cyclic monomers and/or oligomers, optionally in the presence of polymers prone to melt phase transreactions, with reinforcement consisting of high modulus and strength fibers.	[168]

**Table 4:** Preparation and properties of textile reinforced composites via in situ AROP.

Reinforcement	Monomer	Initiator/Activator (Amount)	Technology	Production Parameters	Testing	Results, Comments	Refs.
GF 8-harness satin weave, 300 gsm, E-glass	CL	C1/C20 1.2 mol %/1.2 mol %	VARTM	*P*_p_ = 250 mbar *t*_cycle_ = 60 min *T*_m_ = 110 °C *T*_mold_ = 160 °C	DOC, X_C_, ILSS, ultrasonic analysis, microscopy, VC, mechanical tests	The highest X_C_ = 41% and DOC = 96% were achieved at *T*_mold_ = 160 °C with 6% void content (VC) and ILSS of 62 MPa. *V*_f_ = 50%. The highest ILSS ≈ 68 MPa and the lowest VC = 2% were achieved at *T*_mold_ = 180 °C with a DOC = 93% and the X_C_ = 32% respectively. *V*_f_ = 50%. In both cases a special aminosilane sizing was used. Melt degassing in a buffer vessel.	[169,173]
				*P*_P_ = 250 mbar *t*_cycle_ = 60 min *T*_m_ = 110 °C *T*_mold_ = 180 °C			
				*P*_P_ = 250 mbar *t*_cycle_ = 60 min *T*_m_ = 110 °C *T*_mold_ = 170 °C		Mechanical characteristics were measured in dry as molded, and 23 °C/50% RH conditions: Compressive strength, modulus and strain; Tensile strength, modulus and strain; Shear strength, modulus;	[172]
GF—plain woven S-glass, 400 gsm	CL	C10/C20 1–3/0.5–1.5 mol % molar ratio 2:1	VARTM	C20: 0.5–1.5 mol % *t*_polym_ = 60 min *T*_mold_ = 180 °C	*M*_v_, X_C_, mechanical tests, morphology, ILSS	*M*_v_: 10–12 kDa ILSS: 33–43 MPa Tensile strength: 328–434 MPa Flexural strength: 320–407 MPa; X_C_: 37%–43%	[176]
				C20 = const *t*_polym_ = 60 min *T*_mold_ = 150–190 °C		*M*_v_: 10–12 kDa ILSS: 38–44 MPa Tensile strength: 363–434 MPa Flexural strength: 333–396 MPa; X_C_: 45%–40%	
				C20 = const *t*_polym_ = 5–120 min *T*_mold_ = 160 °C		*M*_v_—almost unchanged ILSS: 38–44 MPa Tensile strength: 382–437 MPa; Flexural strength: 364–395 MPa; X_C_: 41%–44%	
GF-plain weave, 588 gsm, E-glass	CL	C1/4,4′-methylenediphenyl diisocyanate 5/0.9 wt %	VARTM	*t*_cycle_ = 60 min *T*_m_ = 120 °C *T*_mold_ = 160 °C	Microscopy, ^1^H-NMR, FTIR, TGA, DOC	A single-stream processing technique was introduced. An organosilane activator was deposited on the GF surface (*N*-[5-(trimethoxysilyl)-2-aza-1-oxopentyl]caprolactam), and different isocyanate-based activators used. DOC: inlet and outlet: close to 100%, middle: below 25%.	[179]
GF—continuous strand mat (swirl mat), 450 gsm	NBC	-	SRIM	-	Acoustic emission, mechanical tests, IS, microscopy	Fracture toughness (*K*_C_) improved with increasing of *V_f_* of GF. Increasing of the crosshead speed resulted in increased *Kc* that is untypical. IS went through a maximum as a function of temperature. Failure sequence analysis was performed using AE and optical microscopy simultaneously. Fracture mechanics data depended on specimen size and type: the reasonable ligament width and length to span ratio were defined as >12 and >1.7 respectively.	[184,185,186]
CF—4 harness satin weave, 200 gsm	CL	C10/C20 3/1.5 mol %	VARTM	*P*_p_ = 98 kPa *T*_m_ = 100 °C *T*_mold1_ = 100 °C *T*_mold2_ = 150 °C	TGA, DSC, DOC	The mold and the melt temperatures were 100 °C. After a complete impregnation of a preform the temperature was raised up to 150 °C. Average DOC = 98.01%. Average X_C_ = 40%. Reinforcement’s weight fraction, *W*_f_ = 64% (uniform).	[177]
				*P*_p_ = 98 kPa *T*_m_ = 150 °C *T*_mold_ = 150 °C		The mold and melt temperatures were 150 °C. Infusion was incomplete (75% impregnation) due to fast polymerization of the melt. Average DOC = 97.98%. Average X_C_ = 36% (more consistent). *W*_f_ = 52%.	
CF—2/2 twill fabric	LL	NaH/*N,N′*-ethylenebisstearmide	T-RTM	*T*_m_ = 160 °C *P*_clamp_ = 10 bar *T*_mold_ = 270 °C *t*_cycle_ = 10 min	SEM, TGA, density, X_C_, mechanical tests, DMTA	*V*_f_ = 30% Flexural strength = 311 MPa; Flexural modulus = 21.2 GPa; X_C_ = 29% Residual monomer content = 0.9% System working reliably.	[187]
		Grilonit LA 2.5 wt %				*V*_f_ = 54%; Flexural strength = 321.2 MPa; Flexural modulus = 37.8 GPa; X_C_ = 52%.	
CF—woven 2/2 twill, 240 gsm	LL	Grilonit LA 1.5–5 wt %	T-RTM	*P*_P_ = 1 bar (over) *T*_mold_ = 180–250 °C LA = 2 wt %	Mechanical tests, ILSS	*t*_polym_ = 5 min at 250 °C. Processing window: 20 min at 200 °C and 5 min at 250 °C. Tensile strength and stiffness of epoxy based and PA12 based laminates were compared; Testing in ILSS demonstrated plastic deformation instead of failure.	[188]
				*T*_m_ = 180 °C *T*_mold_ = 240 °C *t*_inj_ = 10 sec *t*_polym_ = 8.5 min *P*_P_ = 0.4 bar (over)		Performance of in situ produced PA-12 plate with *V*_f_ = 54% and commingled CF/PA-12 at *V*_f_ = 56% compared. Composites with in situ PA-12 matrix showed good tensile properties under different conditionings while the compressive performance was lower than that of the CF/PA-12 from commingled yarns. ILSS could not be assessed due to plastic deformation of specimens.	[189]
CF—satin weave, 440 gsm	LL	Liquid activating system: NaCL/Carbodiimide 1.5 %	T-RTM	-	Infiltration, diffusion, shrinkage, VC	Matrix shrinkage and residual N_2_ are specified as potential sources of VC growth. At the equilibrium 1.86 kg of N_2_ is dissolved in 1 m^3^ of lactam at 170 °C. Due to pumping of mixture into the mold at 170–190 °C the released gas can generate porosity. To minimize the VC the N_2_ content in the melt should be minimized and an optimal capillary number set for infusion. Bleeding was used to reduce the VC in parts with degassed matrix. The average VC was reduced from 15% to 1%	[174]
CF—5-harness satin weave, 440 gsm	LL	Liquid activator 2–4 %	T-RTM	*T*_m_ = 180 °C *T*_max_ = 255 °C *T*_cool_ = 140 °C *P*_P1_ = 1.5 bar *P*_P2_ = 55 bar *P*_inj_ = 0.2 bar (over) Flow rate = 200 cm^3^/min *t*_cycle_ = 25 min	Mechanical tests, VC	Two types of composites are compared for thermoforming application: commingled CF/PA-12 and in situ polymerized CF/PA-12 Tensile properties were defined before and after heat stamping. *P*_1_—injection/*P*_2_*—*compression ratio varied. Reconsolidation of the composites after preheating remained incomplete. Recycling and overinjection molding strategies presented. VC below 1%.	[181]
NF—ramie, warp/weft yarn 21^S^ × 21^S^, 52 × 36	CL	C1/C20 1.2/0.6 mol %	VARTM	*T*_mold_ = 150 °C *P*_P1_ = 100 mbar	DOC, X_C_, mechanical tests, viscosimetry	DOC = 94.4% X_C_ = 48.0% *M*_v_ = 101 kDa *W*_f_ = 40%.	[178]
		C10/C20 1.2/0.6 mol %		-	FTIR, atomic absorption spectroscopy	Drastic inhibition and discoloration observed with NaOH and C10 initiators in reactive processing due to the byproducts generated by the “peeling reaction” of cellulose in alkaline environment under heat.	

## Abbreviations

^1^H-NMR	proton nuclear magnetic resonance
ABS	acrylonitrile-butadiene-styrene
AE	acoustic emission
AFM	atomic force microscopy
AM	additive manufacturing
AROP	anionic (activated) ring-opening polymerization
C1	Bruggolen C1 (initiator: CLMgBr)
C10	Bruggolen C10 (initiator: NaCL)
C20	Bruggolen C20P (activator: hexamethylene-1.6-dicarbomoylcaprolactam)
CB	carbon black
CBT	cyclic butylene terephthalate
CF	carbon fiber
CL	ε-caprolactam
CLMgBr	ε-caprolactam magnesium bromide
CNC	nanocrystalline cellulose
CNF	carbon nanofibers
CNT	carbon nanotubes
CTT	conversion-temperature transformation
DMA	dynamic mechanical analysis
DMF	dimethylformamide
DOC	degree of conversion
*d*_polym_	length of the polymerization part of the pultrusion die
DSC	differential scanning calorimetry
*E*	Young’s modulus
*F*_pul_	pulling force
FR	flow rate
FTIR	Fourier-transform infrared spectroscopy
*G*	shear modulus
GF	glass fiber
GO	graphene oxide
GPC	gel permeation chromatography
gsm	gram per square meter (surface weight)
HDT	heat distortion temperature
HMDI	hexamethylene di-isocyanate
HSIMT	high speed impact-bending test
ILSS	interlaminar shear strength
IS	impact strength
LCM	liquid composite molding
LDPE	low density polyethylene
LDPE-*g*-MA	LDPE grafted by maleic anhydride
LL	ω-laurolactam
LP-RTM	low pressure resin transfer molding
MA	maleic anhydride
MC	microcapsulation
MMA	methyl methacrylate
MMT	montmorillonite clay
*M*_n_	number average MW
*M*_v_	viscosity average MW
*M*_w_	weight average MW
MW	molecular weight
MWCNT	multiwall carbon nanotube
NaCL	sodium caprolactamate
NBC	nylon block copolymer
NBR	acrylonitrile-butadiene rubber
NMP	*N*-methyl-2-pyrrolidone
NMR	nuclear magnetic resonance
OMMT	organophilic-modified MMT clay
PA	polyamide
PA-12	polyamide-12
PA-6	polyamide-6
PA-6,6	polyamide-6,6
PBT	polybutylene terephthalate
PCL	polycaprolactone
*P*_clamp_	clamping pressure
PDMS	polydimethylsiloxane
phr	parts per hundred parts of resin
PI	polyimide
PIC	phenyl isocyanate (also as cyclic trimer)
*P*_inj_	injection pressure
PMMA	polymethyl methacrylate
POSS	polyhedral oligomeric silsequioxane
PP	polypropylene
*P*_p_	process pressure
PPE	polyphenylene ether
PPG	polypropyleneglycol
PP-*g*-MA	PP grafted by maleic anhydride
PP-*g*-PA-6	PP grafted by PA-6
PS	polystyrene
*R/r*	mold major and minor radius
RH	relative humidity
RIM	reaction injection molding
RISP	reaction-induced phase separation
RMC	residual monomer content
RT	room temperature
RTM	resin transfer molding
SAN	styrene-acrylonitrile
SAXS	small angle X-ray scattering
SCF	short carbon fibers
SEBS	styrene-ethylene-butylene-styrene
SEBS-*g*-MA	SEBS grafted by maleic anhydride
SEC	size exclusion chromatography
SEM	scanning electron microscopy
SMA	styrene-maleic anhydride
SPC	self-reinforced composite
SRIM	structural reaction injection molding
SWCNT	single-walled carbon nanotubes
*T*_0_	initial temperature
*T*_c_	crystallization temperature
*T*_c_	crystallization temperature
*T*_cool_	cooling temperature
*t*_cycle_	cycle time
TDI	toluene 2,4-diisocyanate
TEM	transmission electron microscopy
*T*_f_	fusion temperature
*T*_g_	glass transition temperature
TGA	thermogravimetric analysis
*t*_inj_	injection time
*T*_m_	melting temperature
*T*_max_	process maximum temperature
*T*_mold_	mold temperature
TPC	thermoplastic composites
*t*_polym_	polymerization time
*T*_proc_	processing temperature
TPU	thermoplastic polyurethane
*T*_rc_	recrystallization temperature
TRI-pultrusion	thermoplastic reaction injection pultrusion
TTT	time-temperature-transformation
VC	void content
*V*_f_	fiber (reinforcement) volume content
*V*_pul_	pultrusion line speed
WAXS	wide angle X-ray scattering
*W*_f_	fiber (reinforcement) weight fraction
X_C_	degree of crystallinity
XPS	X-ray photoelectron spectroscopy
XRD	X-ray diffraction
Δ*H*_f_	heat of fusion
ε_R_	elongation at break
η	intrinsic viscosity
μ	viscosity
*ρ*	density
σ_-_	ultimate tensile strength
ω	rotation speed of the rotors
